# The Journal of Cachexia, Sarcopenia and Muscle stays the front‐runner in geriatrics and gerontology

**DOI:** 10.1002/jcsm.12518

**Published:** 2019-12-10

**Authors:** Markus S. Anker, Stefan D. Anker, Andrew J.S. Coats, Stephan von Haehling

**Affiliations:** ^1^ Division of Cardiology and Metabolism, Department of Cardiology Charité Universitätsmedizin Berlin Berlin Germany; ^2^ Berlin Institute of Health Center for Regenerative Therapies (BCRT) Berlin Germany; ^3^ German Centre for Cardiovascular Research (DZHK) partner site Berlin Berlin Germany; ^4^ Department of Cardiology Charité Campus Benjamin Franklin Berlin Germany; ^5^ Department of Cardiology (CVK) Charité Universitätsmedizin Berlin Berlin Germany; ^6^ Charité Universitätsmedizin Berlin Berlin Germany; ^7^ IRCCS San Raffaele Rome Italy; ^8^ Department of Cardiology and Pneumology, Heart Center Göttingen University of Göttingen Medical Center, Georg‐August‐University Göttingen Germany; ^9^ German Center for Cardiovascular Medicine (DZHK), partner site Göttingen Göttingen Germany

The Journal of Cachexia, Sarcopenia and Muscle (JCSM) is an international, peer‐reviewed journal that is published together with the Society on Sarcopenia, Cachexia and Wasting Disorders and with the support of Wiley publishing. Since JCSM is an open‐access journal, all articles are immediately available for free to the entire scientific community. JCSM is devoted to promoting research on cachexia and sarcopenia in chronic illnesses. Other main interests include physiological and pathophysiological changes in body composition in an aging population with and without underlying illness. More recently, a number of publications have also covered the intriguing area of neuromuscular disorders, and Professor Jens Schmidt has joined the editorial team with an excellent knowledge of the area.^1^, ^2^, ^3^, ^4^, ^5^, ^6^, ^7^, ^8^ Special research interests otherwise include lipolysis, muscle wasting, and biomarkers for metabolic changes. The Journal is therefore attractive for many different medical specialities such as clinicians, physicians, trialists, basic scientists, pharmacologists, nurses, physiotherapists, biochemists, biologists, dieticians, and students. Editor‐in‐chief is Professor Stefan D. Anker, co‐editor‐in‐chief is Professor Stephan von Haehling, and senior consulting editor is Professor Andrew J. S. Coats. The editorial team is composed of Monika Diek and Corinna Denecke, which we very much want to thank for their great work. We also want to thank our many different associate editors and reviewers, as well as the authors themselves that constantly submit new papers. JCSM was first published in 2010 and is now in its 10th issue. The number of issues has steadily increased over the years from 2 since 2010, to 4 since 2011, to 5 since 2016, to 6 since 2017, and lastly to 7 since 2018.

Worldwide, all journals are constantly comparing each other with the help of different scores and ratings. In Europe and the United States, the most important rating is the Thomson Scientific impact factor. It is calculated by adding up all citations that are made in the current year for articles published in the last 2 years, divided by the number of original articles and reviews published in the last 2 years. Therefore, the impact factor is always published about 6–7 months after the end of each year—for instance, in summer 2019, the 2018 impact factors were released. For the second time in a row, JCSM has received a two‐digit impact factor: 10.754 (2018), which we think is a tremendous accomplishment (*Figure* 1). Since 2013, JCSM has managed to increase its impact factor by 45%. For comparison, we looked at two other journals that also publish in the fields of cachexia, nutrition, and aging associated changes in the body: ‘Nutrition’ (2018 impact factor 3.591) and ‘The Journal of Nutrition, Health and Aging’ (JNHA, 2018 impact factor 2.660). Since 2013, Nutrition was able to increase its impact factor by 18%, while the impact factor of JNHA remained constant.

**Figure 1:**
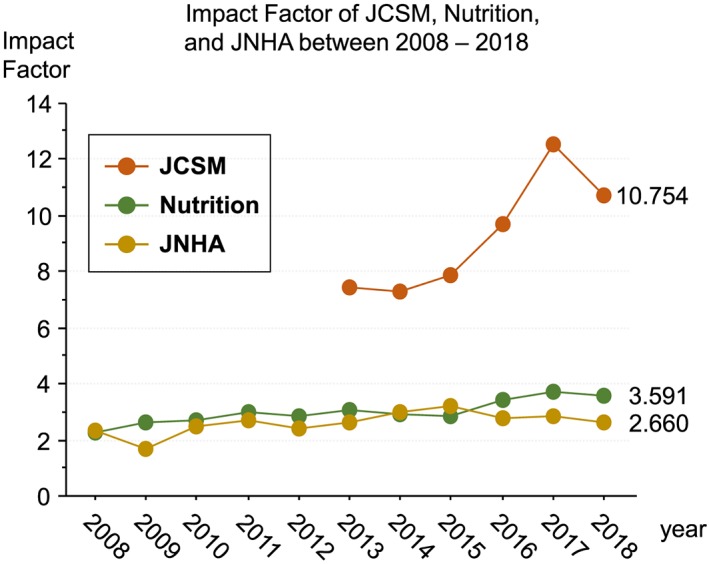
Impact factor of JCSM, Nutrition, and JNHA between 2008 and 2018.

Looking at the most cited scientific papers in JCSM from 2016, 2017, and 2018^9^ (*Tables* 1, 2, 3), one can see that there is great interest in original articles and reviews but also some of the Editorials gather a lot on interest. A total of 33 scientific papers published between 2016 and 2018 have already been cited ≥25 times (counted until 16th of August 2019). In the same time, in the journal Nutrition, a total of 25 scientific papers have been cited ≥25 times (*Tables* 4, 5, 6), while six scientific papers in JNHA have been cited ≥25 times (*Tables* 7, 8, 9). We also looked at the top cited scientific papers ever published in the three journals (*Tables* 10, 11, 12) and found that on average, the top 10 papers in JCSM were cited 152 times, in Nutrition 670 times, and in JNHA 406 times—which is mainly due to the fact that Nutrition has been listed in Scopus^265^ since 1987 and JNHA since 1997, while JCSM started only in 2010. So far, JCSM has published 563 papers, Nutrition 6198 papers, and JNHA 2396 papers. On average, JCSM has published 56 papers/year, Nutrition 188 papers/year, and JNHA 104 papers/year, which of course are major determinants of the respective journal's impact factor. This also underscores that all three journals have different approaches towards the number of papers published per year. We are grateful and look forward to more submissions of excellent research in the field of wasting and muscle disorders and are confident to maintain high quality in the Journal.

**Table 1:** Top 25 scientific publications published in 2016 in the Journal of Cachexia, Sarcopenia and Muscle

Nr.	First author	Title	Type	Times cited	Reference
1	Malmstrom TK	SARC‐F: a symptom score to predict persons with sarcopenia at risk for poor functional outcomes	Original article	105	10
2	Montano‐Loza AJ	Sarcopenic obesity and myosteatosis are associated with higher mortality in patients with cirrhosis	Original article	110	11
3	Anker SD	Welcome to the ICD‐10 code for sarcopenia	Editorial	82	12
4	Coats AJS	Espindolol for the treatment and prevention of cachexia in patients with stage III/IV non‐small cell lung cancer or colorectal cancer: a randomized, double‐blind, placebo‐controlled, international multicentre phase II study (the ACT‐ONE trial)	Original article	67	13
5	Brown JC	Sarcopenia and mortality among a population‐based sample of community‐dwelling older adults	Original article	65	14
6	von Haehling S	Prevalence and clinical impact of cachexia in chronic illness in Europe, USA, and Japan: facts and numbers update 2016	Editorial	56	15
7	Rutten IJG	Loss of skeletal muscle during neoadjuvant chemotherapy is related to decreased survival in ovarian cancer patients	Original article	54	16
8	Tyrovolas S	Factors associated with skeletal muscle mass, sarcopenia, and sarcopenic obesity in older adults: a multi‐continent study	Original article	51	17
9	Leong DP	Reference ranges of handgrip strength from 125,462 healthy adults in 21 countries: a prospective urban rural epidemiologic (PURE) study	Original article	44	18
10	Loncar G	Cardiac cachexia: hic et nunc	Review	41	19
11	Sanders KJC	Cachexia in chronic obstructive pulmonary disease: new insights and therapeutic perspective	Review	39	20
12	Barbosa‐Silva TG	Prevalence of sarcopenia among community‐dwelling elderly of a medium‐sized South American city: results of the COMO VAI? study	Original article	37	21
13	Foong YC	Accelerometer‐determined physical activity, muscle mass, and leg strength in community‐dwelling older adults	Original article	31	22
14	Sente T	Adiponectin resistance in skeletal muscle: pathophysiological implications in chronic heart failure	Review	30	23
15	Sakuma K	p62/SQSTM1 but not LC3 is accumulated in sarcopenic muscle of mice	Original article	29	24
15	Batista ML	Cachexia‐associated adipose tissue morphological rearrangement in gastrointestinal cancer patients	Original article	29	25
17	Patel MS	Growth differentiation factor‐15 is associated with muscle mass in chronic obstructive pulmonary disease and promotes muscle wasting in vivo	Original article	26	26
18	Banach M	Discussion around statin discontinuation in older adults and patients with wasting diseases	Editorial	25	27
18	de Vries NM	Patient‐centred physical therapy is (cost‐) effective in increasing physical activity and reducing frailty in older adults with mobility problems: a randomized controlled trial with 6 months follow‐up	Original article	25	28
18	Lewis A	Increased expression of H19/miR‐675 is associated with a low fat‐free mass index in patients with COPD	Original article	25	29
18	Giron MD	Conversion of leucine to ‐hydroxy‐‐methylbutyrate by ‐keto isocaproate dioxygenase is required for a potent stimulation of protein synthesis in L6 rat myotubes	Original article	25	30
22	Nederveen JP	Skeletal muscle satellite cells are located at a closer proximity to capillaries in healthy young compared with older men	Original article	24	31
22	Go SI	Prognostic impact of sarcopenia in patients with diffuse large B‐cell lymphoma treated with rituximab plus cyclophosphamide, doxorubicin, vincristine, and prednisone	Original article	24	32
22	Pinto CL	Impact of creatine supplementation in combination with resistance training on lean mass in the elderly	Original article	24	33
22	Berger D	Dysfunction of respiratory muscles in critically ill patients on the intensive care unit	Review	24	34

**Table 2:** Top 25 scientific publications published in 2017 in the Journal of Cachexia, Sarcopenia and Muscle

Nr.	First author	Title	Type	Times cited	Reference
1	Kalafateli M	Malnutrition and sarcopenia predict post‐liver transplantation outcomes independently of the Model for End‐stage Liver Disease score	Original article	59	35
2	Solheim TS	A randomized phase II feasibility trial of a multimodal intervention for the management of cachexia in lung and pancreatic cancer	Original article	48	36
3	van Dijk DPJ	Low skeletal muscle radiation attenuation and visceral adiposity are associated with overall survival and surgical site infections in patients with pancreatic cancer	Original article	41	37
4	Boengler K	Mitochondria and ageing: role in heart, skeletal muscle and adipose tissue	Review	36	38
5	Mochamat	A systematic review on the role of vitamins, minerals, proteins, and other supplements for the treatment of cachexia in cancer: a European Palliative Care Research Centre cachexia project	Review	32	39
6	Rutten IJG	Psoas muscle area is not representative of total skeletal muscle area in the assessment of sarcopenia in ovarian cancer	Original article	29	40
7	Brown JL	Mitochondrial degeneration precedes the development of muscle atrophy in progression of cancer cachexia in tumour‐bearing mice	Original article	28	41
8	Nijholt W	The reliability and validity of ultrasound to quantify muscles in older adults: a systematic review	Review	27	42
8	Morley JE	Anorexia of ageing: a key component in the pathogenesis of both sarcopenia and cachexia	Editorial	27	43
8	Snijders T	Muscle fibre capillarization is a critical factor in muscle fibre hypertrophy during resistance exercise training in older men	Original article	27	44
11	Martone AM	The incidence of sarcopenia among hospitalized older patients: results from the Glisten study	Original article	26	45
11	Holecek M	Beta‐hydroxy‐beta‐methylbutyrate supplementation and skeletal muscle in healthy and muscle‐wasting conditions	Review	26	46
11	van Vugt JLA	A comparative study of software programmes for cross‐sectional skeletal muscle and adipose tissue measurements on abdominal computed tomography scans of rectal cancer patients	Original article	26	47
14	Nishikawa H	Elevated serum myostatin level is associated with worse survival in patients with liver cirrhosis	Original article	25	48
14	Lipina C	Lipid modulation of skeletal muscle mass and function	Review	25	49
14	Sahebkar A	Curcumin: an effective adjunct in patients with statin‐associated muscle symptoms?	Review	25	50
17	St‐Jean‐Pelletier F	The impact of ageing, physical activity, and pre‐frailty on skeletal muscle phenotype, mitochondrial content, and intramyocellular lipids in men	Original article	24	51
18	dos Santos L	Sarcopenia and physical independence in older adults: the independent and synergic role of muscle mass and muscle function	Original article	23	52
19	Baracos VE	Psoas as a sentinel muscle for sarcopenia: a flawed premise	Editorial	22	53
19	Gonzalez MC	Bioelectrical impedance analysis for diagnosing sarcopenia and cachexia: what are we really estimating?	Editorial	22	54
19	Klassen O	Muscle strength in breast cancer patients receiving different treatment regimes	Original article	22	55
19	Dodds RM	Prevalence and incidence of sarcopenia in the very old: findings from the Newcastle 85+ Study	Original article	22	56
19	Kittiskulnam P	Sarcopenia among patients receiving hemodialysis: weighing the evidence	Original article	22	57
24	van de Bool C	A randomized clinical trial investigating the efficacy of targeted nutrition as adjunct to exercise training in COPD	Original article	21	58
24	Beaudart C	Validation of the SarQoL, a specific health‐related quality of life questionnaire for Sarcopenia	Original article	21	59

**Table 3:** Top 25 scientific publications published in 2018 in the Journal of Cachexia, Sarcopenia and Muscle

Nr.	First author	Title	Type	Times cited	Reference
1	Buckinx F	Pitfalls in the measurement of muscle mass: a need for a reference standard	Original article	50	60
2	Tieland M	Skeletal muscle performance and ageing	Review	24	61
3	Daly LE	Loss of skeletal muscle during systemic chemotherapy is prognostic of poor survival in patients with foregut cancer	Original article	18	62
3	Choi MH	Sarcopenia is negatively associated with long‐term outcomes in locally advanced rectal cancer	Original article	18	63
5	Rhee CM	Low‐protein diet for conservative management of chronic kidney disease: a systematic review and meta‐analysis of controlled trials	Original article	14	64
6	Zhang ZK	A newly identified lncRNA MAR1 acts as a miR‐487b sponge to promote skeletal muscle differentiation and regeneration	Original article	13	65
6	Muecke M	Systematic review and meta‐analysis of cannabinoids in palliative medicine	Review	13	66
8	Mayr R	Sarcopenia as a comorbidity‐independent predictor of survival following radical cystectomy for bladder cancer	Original article	12	67
8	Calder PC	Targeted medical nutrition for cachexia in chronic obstructive pulmonary disease: a randomized, controlled trial	Original article	12	68
9	Yang QJ	Serum and urine metabolomics study reveals a distinct diagnostic model for cancer cachexia	Original article	11	69
11	Zhang A	miRNA‐23a/27a attenuates muscle atrophy and renal fibrosis through muscle‐kidney crosstalk	Original article	10	70
11	Connolly M	miR‐424‐5p reduces ribosomal RNA and protein synthesis in muscle wasting	Original article	10	71
11	Paul R	miR‐422a suppresses SMAD4 protein expression and promotes resistance to muscle loss	Original article	10	72
14	Ni Bhuachalla EB	Computed tomography diagnosed cachexia and sarcopenia in 725 oncology patients: is nutritional screening capturing hidden malnutrition?	Original article	9	73
14	Cala MP	Multiplatform plasma fingerprinting in cancer cachexia: a pilot observational and translational study	Original article	9	74
14	Hardee JP	Inflammatory signalling regulates eccentric contraction‐induced protein synthesis in cachectic skeletal muscle	Original article	9	75
17	Nissinen TA	Treating cachexia using soluble ACVR2B improves survival, alters mTOR localization, and attenuates liver and spleen responses	Original article	8	76
17	Siracusa J	Circulating myomiRs: a new class of biomarkers to monitor skeletal muscle in physiology and medicine	Review	8	77
19	Kays JK	Three cachexia phenotypes and the impact of fat‐only loss on survival in FOLFIRINOX therapy for pancreatic cancer	Original article	7	78
19	Talbert EE	Circulating monocyte chemoattractant protein‐1 (MCP‐1) is associated with cachexia in treatment‐naive pancreatic cancer patients	Original article	7	79
21	Ebadi M	Poor performance of psoas muscle index for identification of patients with higher waitlist mortality risk in cirrhosis	Original article	6	80
21	Golan T	LY2495655, an antimyostatin antibody, in pancreatic cancer: a randomized, phase 2 trial	Original article	6	81
21	van der Pijl R	Titin‐based mechanosensing modulates muscle hypertrophy	Original article	6	82
21	Peng LN	Healthy community‐living older men differ from women in associations between myostatin levels and skeletal muscle mass	Original article	6	83
21	Shankaran M	Dilution of oral D‐3‐Creatine to measure creatine pool size and estimate skeletal muscle mass: development of a correction algorithm	Original article	6	84

**Table 4:** Top 25 scientific publications published in 2016 in Nutrition

Nr.	First author	Title	Type	Times cited	Reference
1	Akkasheh G	Clinical and metabolic response to probiotic administration in patients with major depressive disorder: a randomized, double‐blind, placebo‐controlled trial	Applied nutritional investigation	112	85
2	Diaz‐Gerevini GT	Beneficial action of resveratrol: how and why?	Review	83	86
3	Sahebkar A	Lipid‐modifying effects of nutraceuticals: an evidence‐based approach	Review	80	87
4	Liu X	Fruit and vegetable consumption and the risk of depression: a meta‐analysis	Review	64	88
5	Hamaguchi Y	Proposal for new diagnostic criteria for low skeletal muscle mass based on computed tomography imaging in Asian adults	Applied nutritional investigation	46	89
6	Obih C	Specific carbohydrate diet for pediatric inflammatory bowel disease in clinical practice within an academic IBD center	Applied nutritional investigation	45	90
7	Venturelli S	Prenylated chalcones and flavonoids for the prevention and treatment of cancer	Review	44	91
8	Sadeghian M	Vitamin D status in relation to Crohn's disease: meta‐analysis of observational studies	Review & meta‐analysis	33	92
8	Thomas MN	Effects of malnutrition on complication rates, length of hospital stay, and revenue in elective surgical patients in the G‐DRG‐system	Applied nutritional investigation	33	93
10	Panahi Y	Effects of supplementation with curcumin on serum adipokine concentrations: a randomized controlled trial	Applied nutritional investigation	32	94
10	Kashtanova DA	Association between the gut microbiota and diet: fetal life, early childhood, and further life	Review	32	95
12	Rouhani MH	Associations between dietary energy density and obesity: a systematic review and meta‐analysis of observational studies	Review	31	96
12	Sarrafzadegan N	Magnesium status and the metabolic syndrome: a systematic review and meta‐analysis	Review	31	97
14	Yamagishi S	Pathologic role of dietary advanced glycation end products in cardiometabolic disorders, and therapeutic intervention	Review	29	98
14	Sahebkar A	Effect of garlic on plasma lipoprotein(a) concentrations: a systematic review and meta‐analysis of randomized controlled clinical trials	Meta‐analysis	29	99
16	Rincon‐Cervera MA	Supplementation with antioxidant‐rich extra virgin olive oil prevents hepatic oxidative stress and reduction of desaturation capacity in mice fed a high‐fat diet: effects on fatty acid composition in liver and extrahepatic tissues	Basic nutritional investigation	28	100
17	Bernini LJ	Beneficial effects of Bifidobacterium lactis on lipid profile and cytokines in patients with metabolic syndrome: a randomized trial. Effects of probiotics on metabolic syndrome	Brief report	27	101
17	Manna P	Beneficial role of vitamin K supplementation on insulin sensitivity, glucose metabolism, and the reduced risk of type 2 diabetes: a review	Review	27	102
19	Schollenberger AE	Impact of protein supplementation after bariatric surgery: a randomized controlled double‐blind pilot study	Applied nutritional investigation	25	103
20	Bounoure L	Detection and treatment of medical inpatients with or at‐risk of malnutrition: suggested procedures based on validated guidelines	Applied nutritional investigation	23	104
21	Sandini M	A high visceral adipose tissue‐to‐skeletal muscle ratio as a determinant of major complications after pancreatoduodenectomy for cancer	Applied nutritional investigation	22	105
21	Marques‐Rocha JL	Expression of inflammation‐related miRNAs in white blood cells from subjects with metabolic syndrome after 8 wk of following a Mediterranean diet‐based weight loss program	Applied nutritional investigation	22	106
23	Caccialanza R	Awareness and consideration of malnutrition among oncologists: insights from an exploratory survey	Brief report	21	107
23	Silvester JA	Is it gluten‐free? Relationship between self‐reported gluten‐free diet adherence and knowledge of gluten content of foods	Applied nutritional investigation	21	108
23	Alvarez JA	Body composition and lung function in cystic fibrosis and their association with adiposity and normal‐weight obesity	Applied nutritional investigation	21	109

**Table 5:** Top 25 scientific publications published in 2017 in Nutrition

Nr.	First author	Title	Type	Times cited	Reference
1	Skalickova S	Selenium nanoparticles as a nutritional supplement	Review	62	110
2	Bjorklund G	Role of oxidative stress and antioxidants in daily nutrition and human health	Review	59	111
3	Sharma K	Converting citrus wastes into value‐added products: economic and environmently friendly approaches	Review	53	112
4	Friedli N	Revisiting the refeeding syndrome: results of a systematic review	Review	44	113
5	DeBoer MD	Systemic inflammation, growth factors, and linear growth in the setting of infection and malnutrition	Applied nutritional investigation	28	114
6	Kaido T	Effects of pretransplant sarcopenia and sequential changes in sarcopenic parameters after living donor liver transplantation	Applied nutritional investigation	24	115
6	Farinetti A	Mediterranean diet and colorectal cancer: a systematic review	Review	24	116
8	Muros JJ	Mediterranean diet adherence is associated with lifestyle, physical fitness, and mental wellness among 10‐y‐olds in Chile	Applied nutritional investigation	22	117
8	Sur S	Molecular aspects of cancer chemopreventive and therapeutic efficacies of tea and tea polyphenols	Review	22	118
8	Eglseer D	Is the presence of a validated malnutrition screening tool associated with better nutritional care in hospitalized patients?	Applied nutritional investigation	22	119
11	Charytoniuk T	Alternative treatment methods attenuate the development of NAFLD: a review of resveratrol molecular mechanisms and clinical trials	Review	18	120
11	Akhtar N	Inhibition of cartilage degradation and suppression of PGE(2) and MMPs expression by pomegranate fruit extract in a model of posttraumatic osteoarthritis	Basic nutritional investigation	18	121
11	Holecek M	Branched‐chain amino acid supplementation in treatment of liver cirrhosis: updated views on how to attenuate their harmful effects on cataplerosis and ammonia formation	Review	18	122
14	Gundala NKV	Arachidonic acid and lipoxinA4 attenuate streptozotocin‐induced cytotoxicity to RIN5 F cells in vitro and type 1 and type 2 diabetes mellitus in vivo	Basic nutritional investigation	17	123
15	Tang Y	Administration of probiotic mixture DM#1 ameliorated 5‐fluorouracil‐induced intestinal mucositis and dysbiosis in rats	Basic nutritional investigation	15	124
15	Abdulrazaq M	Effect of omega‐3 polyunsaturated fatty acids on arthritic pain: a systematic review	Review	15	125
17	Della Corte C	Good adherence to the Mediterranean diet reduces the risk for NASH and diabetes in pediatric patients with obesity: the results of an Italian Study	Applied nutritional investigation	14	126
17	Rajizadeh A	Effect of magnesium supplementation on depression status in depressed patients with magnesium deficiency: a randomized, double‐blind, placebo‐controlled trial	Applied nutritional investigation	14	127
17	Karuppagounder V	Tiny molecule, big power: multi‐target approach for curcumin in diabetic cardiomyopathy	Review	14	128
20	Han S	Lipolysis and thermogenesis in adipose tissues as new potential mechanisms for metabolic benefits of dietary fiber	Basic nutritional investigation	13	129
20	Netto BDM	Eating patterns and food choice as determinant of weight loss and improvement of metabolic profile after RYGB	Applied nutritional investigation	13	130
22	Cruz KJC	Role of microRNAs on adipogenesis, chronic low‐grade inflammation, and insulin resistance in obesity	Review	12	131
22	Clayton ZS	Egg consumption and heart health: a review	Review	12	132
22	Bhaswant M	Anthocyanins in chokeberry and purple maize attenuate diet‐induced metabolic syndrome in rats	Basic nutritional investigation	12	133
22	Aoe S	Effects of high beta‐glucan barley on visceral fat obesity in Japanese individuals: a randomized, double‐blind study	Applied nutritional investigation	12	134

**Table 6:** Top 25 scientific publications published in 2018 in Nutrition

Nr.	First author	Title	Type	Times cited	Reference
1	Schumann D	Low fermentable, oligo‐, di‐, mono‐saccharides and polyol diet in the treatment of irritable bowel syndrome: a systematic review and meta‐analysis	Review	29	135
2	Nowinski A	Trimethylamine N‐oxide: a harmful, protective or diagnostic marker in lifestyle diseases?	Review	17	136
2	Gioxari A	Intake of omega‐3 polyunsaturated fatty acids in patients with rheumatoid arthritis: a systematic review and meta‐analysis	Review	17	137
4	Parker EA	Probiotics and gastrointestinal conditions: an overview of evidence from the Cochrane Collaboration	Review	14	138
5	Tewari N	A comparison of three methods to assess body composition	Applied nutritional investigation	13	139
6	Mafra D	Red meat intake in chronic kidney disease patients: two sides of the coin	Review	11	140
7	Shivappa N	Association of proinflammatory diet with low‐grade inflammation: results from the Moli‐sani study	Applied nutritional investigation	10	141
8	Gianfredi V	Can chocolate consumption reduce cardio‐cerebrovascular risk? A systematic review and meta‐analysis	Review	9	142
8	Zhang N	Time for food: the impact of diet on gut microbiota and human health	Review	9	143
10	Sampasa‐Kanyinga H	Sleep duration and consumption of sugar‐sweetened beverages and energy drinks among adolescents	Applied nutritional investigation	8	144
10	Thiennimitr P	Lactobacillus paracasei HII01, xylooligosaccharides, and synbiotics reduce gut disturbance in obese rats	Basic nutritional investigation	8	145
10	Pineda‐Juarez JA	Body composition evaluated by body mass index and bioelectrical impedance vector analysis in women with rheumatoid arthritis	Applied nutritional investigation	8	146
13	Rinninella E	NutriCatt protocol in the Enhanced Recovery After Surgery (ERAS) program for colorectal surgery: the nutritional support improves clinical and cost‐effectiveness outcomes	Applied nutritional investigation	7	147
13	Bermudes ACG	Changes in lipid metabolism in pediatric patients with severe sepsis and septic shock	Applied nutritional investigation	7	148
15	Mou D	Maternal methyl donor supplementation during gestation counteracts bisphenol A‐induced oxidative stress in sows and offspring	Basic nutritional investigation	6	149
15	Bielinska K	High salt intake increases plasma trimethylamine N‐oxide (TMAO) concentration and produces gut dysbiosis in rats	Basic nutritional investigation	6	150
17	Reichenberger J	It's craving time: time of day effects on momentary hunger and food craving in daily life	Applied nutritional investigation	5	151
17	Brasil GA	The benefits of soluble non‐bacterial fraction of kefir on blood pressure and cardiac hypertrophy in hypertensive rats are mediated by an increase in baroreflex sensitivity and decrease in angiotensin‐converting enzyme activity	Basic nutritional investigation	5	152
17	Ylinen E	Intestinal failure as a significant risk factor for renal impairment in children	Applied nutritional investigation	5	153
17	Kim HM	Caffeic acid ameliorates hepatic steatosis and reduces ER stress in high fat diet‐induced obese mice by regulating autophagy	Basic nutritional investigation	5	154
17	Nunes S	Adherence to a Mediterranean diet and its association with age‐related macular degeneration. The Coimbra Eye Study‐Report 4	Applied nutritional investigation	5	155
22	Moradi S	Associations between dietary inflammatory index and incidence of breast and prostate cancer: a systematic review and meta‐analysis	Review	4	156
22	Shtriker MG	Fenugreek galactomannan and citrus pectin improve several parameters associated with glucose metabolism and modulate gut microbiota in mice	Basic nutritional investigation	4	157
22	Della Valle S	Nutritional intervention in head and neck cancer patients during chemo‐radiotherapy	Brief report	4	158
25	Pounis G	Reduced mortality risk by a polyphenol‐rich diet: an analysis from the Moli‐sani study	Applied nutritional investigation	3	159

**Table 7:** Top 25 scientific publications published in 2016 in The Journal of Nutrition, Health and Aging

Nr.	First author	Title	Type	Times cited	Reference
1	Shimada H	Impact of cognitive frailty on daily activities in older persons	Article	45	160
2	Pilgrim AL	Measuring appetite with the simplified nutritional appetite questionnaire identifies hospitalised older people at risk of worse health outcomes	Article	32	161
3	Boespflug EL	Fish oil supplementation increases event‐related posterior cingulate activation in older adults with subjective memory impairment	Article	27	162
4	Warnier RMJ	Validity, reliability and feasibility of tools to identify frail older patients in inpatient hospital care: a systematic review	Review	25	163
5	Kaehr EW	Frail‐Nh predicts outcomes in long term care	Article	24	164
5	Yoshimura Y	Effects of nutritional supplements on muscle mass and activities of daily living in elderly rehabilitation patients with decreased muscle mass: a randomized controlled trial	Randomised clinical trial	24	165
7	Blain H	A comprehensive fracture prevention strategy in older adults: the European Union Geriatric Medicine Society (EUGMS) statement	Article	22	166
8	Madhavan A	Prevalence of and risk factors for dysphagia in the community dwelling elderly: a systematic review	Review	21	167
9	Tay L	The independent role of inflammation in physical frailty among older adults with mild cognitive impairment and mild‐to‐moderate Alzheimer's disease	Article	20	168
9	Scott D	Associations of low muscle mass and the metabolic syndrome in Caucasian and Asian middle‐aged and older adults	Article	20	169
9	Wakabayashi H	Dysphagia assessed by the 10‐item Eating Assessment Tool is associated with nutritional status and activities of daily living in elderly individuals requiring long‐term care	Article	20	170
12	Armamento‐Villareal R	Effect of lifestyle intervention on the hormonal profile of frail, obese older men	Article	19	171
13	De Vriendt P	Improving health related quality of life and independence in community dwelling frail older adults through a client‐centred and activity‐oriented program. A pragmatic randomized controlled trial	Randomised clinical trial	18	172
13	Vasconcelos KS	Handgrip strength cutoff points to identify mobility limitation in community‐dwelling older people and associated factors	Article	18	173
13	Molino S	Sarcopenic obesity: an appraisal of the current status of knowledge and management in elderly people	Article	18	174
16	Morilla‐Herrera JC	Effectiveness of food‐based fortification in older people a systematic review and meta‐analysis	Review	17	175
17	Martinez‐Velilla N	Physical activity and early rehabilitation in hospitalized elderly medical patients: systematic review of randomized clinical trials	Review	16	176
17	Fougere B	Association between the Mediterranean‐style dietary pattern score and physical performance: results from Trelong study	Article	16	177
19	Abraha I	Non‐pharmacological interventions to prevent or treat delirium in older patients: clinical practice recommendations the SENATOR‐ONTOP series	Article	15	178
19	Hajek A	Predictors of frailty in old age‐results of a longitudinal study	Article	15	179
21	Chode S	Frailty, diabetes, and mortality in middle‐aged African Americans	Article	14	180
21	Hentzien M	Impact of age‐related comorbidities on five‐year overall mortality among elderly HIV‐infected patients in the late HAART era—role of chronic renal disease	Article	14	181
21	Lehtisalo J	Association of long‐term dietary fat intake, exercise, and weight with later cognitive function in the Finnish Diabetes Prevention Study	Article	14	182
24	van Wissen J	Mini nutritional assessment and mortality after hip fracture surgery in the elderly	Article	12	183
25	Beasley JM	Is meeting the recommended dietary allowance (RDA) for protein related to body composition among older adults?: results from the Cardiovascular Health of Seniors and Built Environment Study	Article	10	184

**Table 8:** Top 25 scientific publications published in 2017 in The Journal of Nutrition, Health and Aging

Nr.	First author	Title	Type	Times cited	Reference
1	Wirth MD	Construct validation of the dietary inflammatory index among African Americans	Article	36	185
2	Roppolo M	Cognitive frailty in Italian community‐dwelling older adults: prevalence rate and its association with disability	Article	24	186
3	Balogun S	Prospective associations of low muscle mass and function with 10‐year falls risk, incident fracture and mortality in community‐dwelling older adults	Article	21	187
4	Bousquet J	Building bridges for innovation in ageing: synergies between action groups of the EIP on AHA	Article	20	188
5	Zhang YY	Efficacy of omega‐3 polyunsaturated fatty acids supplementation in managing overweight and obesity: a meta‐analysis of randomized clinical trials	Meta‐analysis	19	189
5	Misciagna G	Effect of a low glycemic index Mediterranean diet on non‐alcoholic fatty liver disease. A randomized controlled clinici trial	Randomised clinical trial	19	190
5	O'Shea E	Malnutrition in hospitalised older adults: a multicentre observational study of prevalence, associations and outcomes	Article	19	191
5	Hooper C	Cognitive changes with omega‐3 polyunsaturated fatty acids in non‐demented older adults with low omega‐3 index	Article	19	192
9	Tieland M	The impact of dietary protein or amino acid supplementation on muscle mass and strength in elderly people: individual participant data and meta‐analysis of RCT's	Meta‐analysis	18	193
10	Limongi F	Adherence to the Mediterranean diet and all‐cause mortality risk in an elderly Italian population: data from the ILSA study	Article	15	194
11	Masanes F	Cut‐off points for muscle mass—not grip strength or gait speed—determine variations in sarcopenia prevalence	Article	14	195
11	Mitchell EL	Reduced intestinal motility, mucosal barrier function, and inflammation in aged monkeys	Article	14	196
13	Landi F	Animal‐derived protein consumption is associated with muscle mass and strength in community‐dwellers: results from the Milan EXPO survey	Article	13	197
13	Amamou T	Effect of a high‐protein energy‐restricted diet combined with resistance training on metabolic profile in older individuals with metabolic impairments	Article	13	198
13	Sargent L	Assessing the current state of cognitive frailty: measurement properties	Article	13	199
16	Iolascon G	Are dietary supplements and nutraceuticals effective for musculoskeletal health and cognitive function? A scoping review	Review	12	200
17	Garcia‐Nogueras I	Use of health resources and healthcare costs associated with frailty: the FRADEA study	Article	11	201
18	Beelen J	Protein enrichment of familiar foods as an innovative strategy to increase protein intake in institutionalized elderly	Article	10	202
18	Fielding RA	Effect of structured physical activity and nutritional supplementation on physical function in mobility‐limited older adults: results from the VIVE2 randomized trial	Article	10	203
18	Dyer J	Effect of a Mediterranean type diet on inflammatory and cartilage degradation biomarkers in patients with osteoarthritis	Article	10	204
21	Tucker LA	Consumption of nuts and seeds and telomere length in 5,582 men and women of the National Health and Nutrition Examination Survey (NHANES)	Article	9	205
22	Bleijenberg N	Disability in the individual ADL, IADL, and mobility among older adults: a prospective cohort study	Article	8	206
22	Chassagne P	Tolerance and long‐term efficacy of polyethylene glycol 4000 (ForlaxA (R)) compared to lactulose in elderly patients with chronic constipation	Article	8	207
24	Harada H	Effectiveness of cardiac rehabilitation for prevention and treatment of sarcopenia in patients with cardiovascular disease—a retrospective cross‐sectional analysis	Article	6	208
25	Ritt M	High‐technology based gait assessment in frail people: associations between spatio‐temporal and three‐dimensional gait characteristics with frailty status across four different frailty measures	Article	4	209

**Table 9:** Top 25 scientific publications published in 2018 in The Journal of Nutrition, Health and Aging

Nr.	First author	Title	Type	Times cited	Reference
1	Dent E	International clinical practice guidelines for sarcopenia (ICFSR): screening, diagnosis and management	Article	27	210
2	Berendsen AM	Association of long‐term adherence to the mind diet with cognitive function and cognitive decline in American women	Article	12	211
3	Marshall S	Why is the skeleton still in the hospital closet? A look at the complex aetiology of protein‐energy malnutrition and its implications for the nutrition care team	Article	9	212
4	McCullough J	The My Meal Intake Tool (M‐MIT): validity of a patient self‐assessment for food and fluid intake at a single meal	Article	9	213
4	Beaudart C	Effects of protein, essential amino acids, B‐hydroxy B‐methylbutyrate, creatine, dehydroepiandrosterone and fatty acid supplementation on muscle mass, muscle strength and physical performance in older people aged 60 years and over. a systematic review of the literature	Review	9	214
6	Rietman ML	The association between BMI and different frailty domains: a U‐shaped curve?	Article	5	215
7	Zhao WT	Systematic review and meta‐analysis of the association between sarcopenia and dysphagia	Review	6	216
7	Kim J	Nutritional status and frailty in community‐dwelling older Korean adults: the Korean Frailty and Aging Cohort Study	Article	5	217
9	Wang T	Usefulness of Simplified Nutritional Appetite Questionnaire (Snaq) in appetite assessment in elder patients with liver cirrhosis	Article	6	218
9	Sanz‐Paris A	Role of oral nutritional supplements enriched with B‐hydroxy‐B‐methylbutyrate in maintaining muscle function and improving clinical outcomes in various clinical settings	Article	6	219
9	Yu Y	Berberine improves cognitive deficiency and muscular dysfunction via activation of the AMPK/SIRT1/PGC‐1a pathway in skeletal muscle from naturally aging rats	Article	5	220
9	Pagliai G	Mediterranean diet, food consumption and risk of late‐life depression: the Mugello study	Article	6	221
9	Munoz‐Gonzalez C	Association between salivary hypofunction and food consumption in the elderlies. A systematic literature review	Review	4	222
9	Hidayat K	Effects of milk proteins supplementation in older adults undergoing resistance training: a meta‐analysis of randomized control trials	Meta‐analysis	5	223
15	Nowson CA	The impact of dietary factors on indices of chronic disease in older people: a systematic review	Review	8	224
15	Eglseer D	Dysphagia in hospitalized older patients: associated factors and nutritional interventions	Article	4	225
17	Derstine BA	Quantifying sarcopenia reference values using lumbar and thoracic muscle areas in a healthy population	Article	3	226
17	EL Hajj C	Effect of vitamin D treatment on glucose homeostasis and metabolism in Lebanese older adults: a randomized controlled trial	Randomised controlled trial	3	227
17	Rodriguez Manas L	Key messages for a frailty prevention and management policy in Europe from the Advantage Joint Action consortium	Article	3	228
17	Tek NA	Determinants of health‐related quality of life in home dwelling elderly population: appetite and nutritional status	Article	5	229
21	Palmer K	The relationship between anaemia and frailty: a systematic review and meta‐analysis of observational studies	Review	5	230
21	Rodriguez‐Rejon AI	Diagnosis of sarcopenia in long‐term care homes for the elderly: the sensitivity and specificity of two simplified algorithms with respect to the EWGSOP consensus	Article	4	231
21	Payne M	Dysphagia, dementia and frailty	Article	4	232
21	Wang Y	Adherence to the Mediterranean diet and the risk of frailty in old people: a systematic review and meta‐analysis	Review	5	233
21	Lim SER	Assessment of physical activity of hospitalised older adults: a systematic review	Review	4	234

**Table 10:** Top 10 scientific publications published in all years in the Journal of Cachexia, Sarcopenia and Muscle

Nr.	First author	Title	Year published	Type	Times cited	Reference
1	von Haehling S	Cachexia as a major underestimated and unmet medical need: facts and numbers	2010	Editorial	392	235
2	Dalton JT	The selective androgen receptor modulator GTx‐024 (enobosarm) improves lean body mass and physical function in healthy elderly men and postmenopausal women: results of a double‐blind, placebo‐controlled phase II trial	2011	Original article	159	236
3	Morley JE	Prevalence, incidence, and clinical impact of sarcopenia: facts, numbers, and epidemiology‐update 2014	2014	Editorial	148	237
3	Fanzani A	Molecular and cellular mechanisms of skeletal muscle atrophy: an update	2012	Review	125	238
5	Cesari M	Biomarkers of sarcopenia in clinical trials‐recommendations from the International Working Group on Sarcopenia	2012	Original article	121	239
6	Lenk K	Skeletal muscle wasting in cachexia and sarcopenia: molecular pathophysiology and impact of exercise training	2015	Review	118	240
7	Wakabayashi H	Rehabilitation nutrition for sarcopenia with disability: a combination of both rehabilitation and nutrition care management	2014	Review	116	241
8	Morley JE	From sarcopenia to frailty: a road less travelled	2014	Editorial	115	242
9	Elkina Y	The role of myostatin in muscle wasting: an overview	2011	Review	115	243
10	von Haehling, Stephan	An overview of sarcopenia: facts and numbers on prevalence and clinical impact	2010	Editorial	109	244

**Table 11:** Top 10 scientific publications published in all years in Nutrition

Nr.	First author	Title	Year published	Type	Times cited	Reference
1	Fang YZ	Free radicals, antioxidants, and nutrition	2002	Regulation of physiological systems by nutrients	1511	245
2	Vellas B	The mini nutritional assessment (MNA) and its use in grading the nutritional state of elderly patients	1999	Applied nutritional investigation	805	246
3	Dubois D	Nutrition Metabolism Classic—A formula to estimate the approximate surface‐area if height and weight be known (Reprinted From Archives Internal Medicine, Vol 17, Pg 863, 1916)	1989	Article	655	247
4	Torres SJ	Relationship between stress, eating behavior, and obesity	2007	Review	573	248
5	Kuhajda FP	Fatty‐acid synthase and human cancer: new perspectives on its role in tumor biology	2000	Review	567	249
6	Das UN	Is obesity an inflammatory condition?	2001	Hypothesis: food for thought	565	250
7	Waterland RA	Early nutrition, epigenetic changes at transposons and imprinted genes, and enhanced susceptibility to adult chronic diseases	2004	Epigenetics and epistasis	563	251
8	Slavin JL	Dietary fiber and body weight	2005	Review	534	252
8	Barker DJ	Maternal nutrition, fetal nutrition, and disease in later life	1997	Review	469	253
10	Scalzo J	Plant genotype affects total antioxidant capacity and phenolic contents in fruit	2005	Basic nutritional investigation	458	254

**Table 12:** Top 10 scientific publications published in all years in The Journal of Nutrition, Health and Aging

Nr.	First author	Title	Year published	Type	Times cited	Reference
1	Abellan van Kan G	Gait speed at usual pace as a predictor of adverse outcomes in community‐dwelling older people an International Academy on Nutrition and Aging (IANA) Task Force	2009	Article	724	255
2	Guigoz Y	The Mini Nutritional Assessment (MNA (R)) review of the literature—what does it tell us?	2006	Review	544	256
3	Kaiser MJ	Validation of the Mini Nutritional Assessment short‐form (MNAA (R)‐SF): a practical tool for identification of nutritional status	2009	Article	511	257
4	Abellan van Kan G	The IANA task force on frailty assessment of older people in clinical practice	2008	Geriatric Science	443	258
5	Rolland Y	Sarcopenia: its assessment, etiology, pathogenesis, consequences and future perspectives	2008	Article	421	259
6	Vellas B	Overview of the MNA (R)—its history and challenges	2006	Article	370	260
7	Morley JE	A simple frailty questionnaire (FRAIL) predicts outcomes in middle aged African Americans	2012	Article	343	261
8	Bourre JM	Effects of nutrients (in food) on the structure and function of the nervous system: update on dietary requirements for brain. Part 1: micronutrients	2006	Article	238	262
9	Jugdaohsingh R	Silicon and bone health	2007	Article	237	263
10	Kelaiditi E	Cognitive frailty: rational and definition from an (IANA/IAGG) international consensus group	2013	Article	231	264

## Conflict of interest

None declared.

## References

[1] Hong Y , Lee JH , Jeong KW , Choi CS , Jun HS . Amelioration of muscle wasting by glucagon‐like peptide‐1 receptor agonist in muscle atrophy. J Cachexia Sarcopenia Muscle 2019;10:903–918. 31020810 10.1002/jcsm.12434 PMC6711418

[2] Hughes MC , Ramos SV , Turnbull PC , Rebalka IA , Cao A , Monaco CMF , et al. Early myopathy in Duchenne muscular dystrophy is associated with elevated mitochondrial H2 O2 emission during impaired oxidative phosphorylation. J Cachexia Sarcopenia Muscle 2019;10:643–661. 30938481 10.1002/jcsm.12405 PMC6596403

[3] Zhang P , He J , Wang F , Gong J , Wang L , Wu Q , et al. Hemojuvelin is a novel suppressor for Duchenne muscular dystrophy and age‐related muscle wasting. J Cachexia Sarcopenia Muscle 2019;10:557–573. 30884219 10.1002/jcsm.12414 PMC6596404

[4] Jacques MF , Onambele‐Pearson GL , Reeves ND , Stebbings GK , Smith J , Morse CI . Relationships between muscle size, strength, and physical activity in adults with muscular dystrophy. J Cachexia Sarcopenia Muscle 2018;9:1042–1052. 30338901 10.1002/jcsm.12347 PMC6240748

[5] González‐Sánchez J , Sánchez‐Temprano A , Cid‐Díaz T , Pabst‐Fernández R , Mosteiro CS , Gallego R , et al. Improvement of Duchenne muscular dystrophy phenotype following obestatin treatment. J Cachexia Sarcopenia Muscle 2018;9:1063–1078. 30216693 10.1002/jcsm.12338 PMC6240759

[6] Spitali P , Hettne K , Tsonaka R , Charrout M , van den Bergen J , Koeks Z , et al. Tracking disease progression non‐invasively in Duchenne and Becker muscular dystrophies. J Cachexia Sarcopenia Muscle 2018;9:715–726. 29682908 10.1002/jcsm.12304 PMC6104105

[7] Walter MC , Reilich P . Recent developments in Duchenne muscular dystrophy: facts and numbers. J Cachexia Sarcopenia Muscle 2017;8:681–685. 29076660 10.1002/jcsm.12245 PMC5659056

[8] Narasimhan A , Greiner R , Bathe OF , Baracos V , Damaraju S . Differentially expressed alternatively spliced genes in skeletal muscle from cancer patients with cachexia. J Cachexia Sarcopenia Muscle 2018;9:60–70. 28984045 10.1002/jcsm.12235 PMC5803615

[9] www.webofknowledge.com.

[10] Malmstrom TK , Miller DK , Simonsick EM , Ferrucci L , Morley JE . SARC‐F: a symptom score to predict persons with sarcopenia at risk for poor functional outcomes. J Cachexia Sarcopenia Muscle 2016;7:28–36. 27066316 10.1002/jcsm.12048 PMC4799853

[11] Montano‐Loza AJ , Angulo P , Meza‐Junco J , Prado CMM , Sawyer MB , Beaumont C , et al. Sarcopenic obesity and myosteatosis are associated with higher mortality in patients with cirrhosis. J Cachexia Sarcopenia Muscle 2016;7:126–135. 27493866 10.1002/jcsm.12039 PMC4864157

[12] Anker SD , Morley JE , Haehling S . Welcome to the ICD‐10 code for sarcopenia. J Cachexia Sarcopenia Muscle 2016;7:512–514. 27891296 10.1002/jcsm.12147 PMC5114626

[13] Stewart Coats AJ , Ho GF , Prabhash K , Haehling S , Tilson J , Brown R , et al. Espindolol for the treatment and prevention of cachexia in patients with stage III/IV non‐small cell lung cancer or colorectal cancer: a randomized, double‐blind, placebo‐controlled, international multicentre phase II study (the ACT‐ONE trial). J Cachexia Sarcopenia Muscle 2016;7:355–365. 27386169 10.1002/jcsm.12126 PMC4929828

[14] Brown JC , Harhay MO , Harhay MN . Sarcopenia and mortality among a population‐based sample of community‐dwelling older adults. J Cachexia Sarcopenia Muscle 2016;7:290–298. 27239410 10.1002/jcsm.12073 PMC4864252

[15] Haehling S , Anker MS , Anker SD . Prevalence and clinical impact of cachexia in chronic illness in Europe, USA, and Japan: facts and numbers update 2016. J Cachexia Sarcopenia Muscle 2016;7:507–509. 27891294 10.1002/jcsm.12167 PMC5114624

[16] Rutten IJG , Dijk DPJ , Kruitwagen RFPM , Beets‐Tan RGH , Olde Damink SWM , Gorp T . Loss of skeletal muscle during neoadjuvant chemotherapy is related to decreased survival in ovarian cancer patients. J Cachexia Sarcopenia Muscle 2016;7:458–466. 27030813 10.1002/jcsm.12107 PMC4782251

[17] Tyrovolas S , Koyanagi A , Olaya B , Ayuso‐Mateos JL , Miret M , Chatterji S , et al. Factors associated with skeletal muscle mass, sarcopenia, and sarcopenic obesity in older adults: a multi‐continent study. J Cachexia Sarcopenia Muscle 2016;7:312–321. 27239412 10.1002/jcsm.12076 PMC4864288

[18] Leong DP , Teo KK , Rangarajan S , Kutty VR , Lanas F , Hui C , et al. Reference ranges of handgrip strength from 125,462 healthy adults in 21 countries: a prospective urban rural epidemiologic (PURE) study. J Cachexia Sarcopenia Muscle 2016;7:535–546. 27104109 10.1002/jcsm.12112 PMC4833755

[19] Loncar G , Springer J , Anker M , Doehner W , Lainscak M . Cardiac cachexia: hic et nunc. J Cachexia Sarcopenia Muscle 2016;7:246–260. 27386168 10.1002/jcsm.12118 PMC4929818

[20] Sanders KJC , Kneppers AEM , Bool C , Langen RCJ , Schols AMWJ . Cachexia in chronic obstructive pulmonary disease: new insights and therapeutic perspective. J Cachexia Sarcopenia Muscle 2016;7:5–22. 27066314 10.1002/jcsm.12062 PMC4799856

[21] Barbosa‐Silva TG , Bielemann RM , Gonzalez MC , Menezes AMB . Prevalence of sarcopenia among community‐dwelling elderly of a medium‐sized South American city: results of the *COMO VAI*? study. J Cachexia Sarcopenia Muscle 2016;7:136–143. 27493867 10.1002/jcsm.12049 PMC4864188

[22] Foong YC , Chherawala N , Aitken D , Scott D , Winzenberg T , Jones G . Accelerometer‐determined physical activity, muscle mass, and leg strength in community‐dwelling older adults. J Cachexia Sarcopenia Muscle 2016;7:275–283. 27239404 10.1002/jcsm.12065 PMC4863829

[23] Sente T , Van Berendoncks AM , Hoymans VY , Vrints CJ . Adiponectin resistance in skeletal muscle: pathophysiological implications in chronic heart failure. J Cachexia Sarcopenia Muscle 2016;7:261–274. 27239409 10.1002/jcsm.12086 PMC4864225

[24] Sakuma K , Kinoshita M , Ito Y , Aizawa M , Aoi W , Yamaguchi A . p62/SQSTM1 but not LC3 is accumulated in sarcopenic muscle of mice. J Cachexia Sarcopenia Muscle 2016;7:204–212. 27493873 10.1002/jcsm.12045 PMC4864138

[25] Batista ML , Henriques FS , Neves RX , Olivan MR , Matos‐Neto EM , Alcântara PSM , et al. Cachexia‐associated adipose tissue morphological rearrangement in gastrointestinal cancer patients. J Cachexia Sarcopenia Muscle 2016;7:37–47. 27066317 10.1002/jcsm.12037 PMC4799865

[26] Patel MS , Lee J , Baz M , Wells CE , Bloch S , Lewis A , et al. Growth differentiation factor‐15 is associated with muscle mass in chronic obstructive pulmonary disease and promotes muscle wasting *in vivo* . J Cachexia Sarcopenia Muscle 2016;7:436–448. 27239406 10.1002/jcsm.12096 PMC4864181

[27] Banach M , Serban M‐C . Discussion around statin discontinuation in older adults and patients with wasting diseases. J Cachexia Sarcopenia Muscle 2016;7:396–399. 27030814 10.1002/jcsm.12109 PMC4782254

[28] Vries NM , Staal JB , Wees PJ , Adang EMM , Akkermans R , Olde Rikkert MGM , et al. Patient‐centred physical therapy is (cost‐) effective in increasing physical activity and reducing frailty in older adults with mobility problems: a randomized controlled trial with 6 months follow‐up. J Cachexia Sarcopenia Muscle 2016;7:422–435. 27239405 10.1002/jcsm.12091 PMC4864107

[29] Lewis A , Lee JY , Donaldson AV , Natanek SA , Vaidyanathan S , Man WD‐C , et al. Increased expression of H19/miR‐675 is associated with a low fat‐free mass index in patients with COPD. J Cachexia Sarcopenia Muscle 2016;7:330–344. 27239417 10.1002/jcsm.12078 PMC4863928

[30] Girón MD , Vílchez JD , Salto R , Manzano M , Sevillano N , Campos N , et al. Conversion of leucine to β‐hydroxy‐β‐methylbutyrate by α‐keto isocaproate dioxygenase is required for a potent stimulation of protein synthesis in L6 rat myotubes. J Cachexia Sarcopenia Muscle 2016;7:68–78. 27065075 10.1002/jcsm.12032 PMC4799859

[31] Nederveen JP , Joanisse S , Snijders T , Ivankovic V , Baker SK , Phillips SM , et al. Skeletal muscle satellite cells are located at a closer proximity to capillaries in healthy young compared with older men. J Cachexia Sarcopenia Muscle 2016;7:547–554. 27239425 10.1002/jcsm.12105 PMC4864218

[32] Go S‐I , Park MJ , Song H‐N , Kim H‐G , Kang MH , Lee HR , et al. Prognostic impact of sarcopenia in patients with diffuse large B‐cell lymphoma treated with rituximab plus cyclophosphamide, doxorubicin, vincristine, and prednisone. J Cachexia Sarcopenia Muscle 2016;7:567–576. 27104110 10.1002/jcsm.12115 PMC4833756

[33] Pinto CL , Botelho PB , Carneiro JA , Mota JF . Impact of creatine supplementation in combination with resistance training on lean mass in the elderly. J Cachexia Sarcopenia Muscle 2016;7:413–421. 27239423 10.1002/jcsm.12094 PMC4864174

[34] Berger D , Bloechlinger S , Haehling S , Doehner W , Takala J , Z'Graggen WJ , et al. Dysfunction of respiratory muscles in critically ill patients on the intensive care unit. J Cachexia Sarcopenia Muscle 2016;7:403–412. 27030815 10.1002/jcsm.12108 PMC4788634

[35] Kalafateli M , Mantzoukis K , Choi Yau Y , Mohammad AO , Arora S , Rodrigues S , et al. Malnutrition and sarcopenia predict post‐liver transplantation outcomes independently of the Model for End‐stage Liver Disease score. J Cachexia Sarcopenia Muscle 2017;8:113–121. 27239424 10.1002/jcsm.12095 PMC4864202

[36] Solheim TS , Laird BJA , Balstad TR , Stene GB , Bye A , Johns N , et al. A randomized phase II feasibility trial of a multimodal intervention for the management of cachexia in lung and pancreatic cancer. J Cachexia Sarcopenia Muscle 2017;8:778–788. 28614627 10.1002/jcsm.12201 PMC5659068

[37] Dijk DPJ , Bakens MJAM , Coolsen MME , Rensen SS , Dam RM , Bours MJL , et al. Low skeletal muscle radiation attenuation and visceral adiposity are associated with overall survival and surgical site infections in patients with pancreatic cancer. J Cachexia Sarcopenia Muscle 2017;8:317–326. 27897432 10.1002/jcsm.12155 PMC5377384

[38] Boengler K , Kosiol M , Mayr M , Schulz R , Rohrbach S . Mitochondria and ageing: role in heart, skeletal muscle and adipose tissue. J Cachexia Sarcopenia Muscle 2017;8:349–369. 28432755 10.1002/jcsm.12178 PMC5476857

[39] Cuhls H , Marinova M , Kaasa S , Stieber C , Conrad R , Radbruch L , et al. A systematic review on the role of vitamins, minerals, proteins, and other supplements for the treatment of cachexia in cancer: a European Palliative Care Research Centre cachexia project. J Cachexia Sarcopenia Muscle 2017;8:25–39. 27897391 10.1002/jcsm.12127 PMC5326814

[40] Rutten IJG , Ubachs J , Kruitwagen RFPM , Beets‐Tan RGH , Olde Damink SWM , Van Gorp T . Psoas muscle area is not representative of total skeletal muscle area in the assessment of sarcopenia in ovarian cancer. J Cachexia Sarcopenia Muscle 2017;8:630–638. 28513088 10.1002/jcsm.12180 PMC5566632

[41] Brown JL , Rosa‐Caldwell ME , Lee DE , Blackwell TA , Brown LA , Perry RA , et al. Mitochondrial degeneration precedes the development of muscle atrophy in progression of cancer cachexia in tumour‐bearing mice. J Cachexia Sarcopenia Muscle 2017;8:926–938. 28845591 10.1002/jcsm.12232 PMC5700433

[42] Nijholt W , Scafoglieri A , Jager‐Wittenaar H , Hobbelen JSM , Schans CP . The reliability and validity of ultrasound to quantify muscles in older adults: a systematic review. J Cachexia Sarcopenia Muscle 2017;8:702–712. 28703496 10.1002/jcsm.12210 PMC5659048

[43] Morley JE . Anorexia of ageing: a key component in the pathogenesis of both sarcopenia and cachexia. J Cachexia Sarcopenia Muscle 2017;8:523–526. 28452130 10.1002/jcsm.12192 PMC5566640

[44] Snijders T , Nederveen JP , Joanisse S , Leenders M , Verdijk LB , Loon LJC , et al. Muscle fibre capillarization is a critical factor in muscle fibre hypertrophy during resistance exercise training in older men. J Cachexia Sarcopenia Muscle 2017;8:267–276. 27897408 10.1002/jcsm.12137 PMC5377411

[45] Martone AM , Bianchi L , Abete P , Bellelli G , Bo M , Cherubini A , et al. The incidence of sarcopenia among hospitalized older patients: results from the Glisten study. J Cachexia Sarcopenia Muscle 2017;8:907–914. 28913934 10.1002/jcsm.12224 PMC5700449

[46] Holeček M . Beta‐hydroxy‐beta‐methylbutyrate supplementation and skeletal muscle in healthy and muscle‐wasting conditions. J Cachexia Sarcopenia Muscle 2017;8:529–541. 28493406 10.1002/jcsm.12208 PMC5566641

[47] Vugt JLA , Levolger S , Gharbharan A , Koek M , Niessen WJ , Burger JWA , et al. A comparative study of software programmes for cross‐sectional skeletal muscle and adipose tissue measurements on abdominal computed tomography scans of rectal cancer patients. J Cachexia Sarcopenia Muscle 2017;8:285–297. 27897414 10.1002/jcsm.12158 PMC5697014

[48] Nishikawa H , Enomoto H , Ishii A , Iwata Y , Miyamoto Y , Ishii N , et al. Elevated serum myostatin level is associated with worse survival in patients with liver cirrhosis. J Cachexia Sarcopenia Muscle 2017;8:915–925. 28627027 10.1002/jcsm.12212 PMC5700437

[49] Lipina C , Hundal HS . Lipid modulation of skeletal muscle mass and function. J Cachexia Sarcopenia Muscle 2017;8:190–201. 27897400 10.1002/jcsm.12144 PMC5377414

[50] Sahebkar A , Saboni N , Pirro M , Banach M . Curcumin: an effective adjunct in patients with statin‐associated muscle symptoms? J Cachexia Sarcopenia Muscle 2017;8:19–24. 27897416 10.1002/jcsm.12140 PMC5326825

[51] St‐Jean‐Pelletier F , Pion CH , Leduc‐Gaudet J‐P , Sgarioto N , Zovilé I , Barbat‐Artigas S , et al. The impact of ageing, physical activity, and pre‐frailty on skeletal muscle phenotype, mitochondrial content, and intramyocellular lipids in men. J Cachexia Sarcopenia Muscle 2017;8:213–228. 27897402 10.1002/jcsm.12139 PMC5377417

[52] Santos L , Cyrino ES , Antunes M , Santos DA , Sardinha LB . Sarcopenia and physical independence in older adults: the independent and synergic role of muscle mass and muscle function. J Cachexia Sarcopenia Muscle 2017;8:245–250. 27897417 10.1002/jcsm.12160 PMC5377449

[53] Baracos VE . *Psoas* as a sentinel muscle for sarcopenia: a flawed premise. J Cachexia Sarcopenia Muscle 2017;8:527–528. 28675689 10.1002/jcsm.12221 PMC5566635

[54] Gonzalez MC , Heymsfield SB . Bioelectrical impedance analysis for diagnosing sarcopenia and cachexia: what are we really estimating? J Cachexia Sarcopenia Muscle 2017;8:187–189. 28145079 10.1002/jcsm.12159 PMC5377383

[55] Klassen O , Schmidt ME , Ulrich CM , Schneeweiss A , Potthoff K , Steindorf K , et al. Muscle strength in breast cancer patients receiving different treatment regimes. J Cachexia Sarcopenia Muscle 2017;8:305–316. 27896952 10.1002/jcsm.12165 PMC5377413

[56] Dodds RM , Granic A , Davies K , Kirkwood TBL , Jagger C , Sayer AA . Prevalence and incidence of sarcopenia in the very old: findings from the Newcastle 85+ Study. J Cachexia Sarcopenia Muscle 2017;8:229–237. 27897431 10.1002/jcsm.12157 PMC5377385

[57] Kittiskulnam P , Carrero JJ , Chertow GM , Kaysen GA , Delgado C , Johansen KL . Sarcopenia among patients receiving hemodialysis: weighing the evidence. J Cachexia Sarcopenia Muscle 2017;8:57–68. 27897415 10.1002/jcsm.12130 PMC5326818

[58] Bool C , Rutten EPA , Helvoort A , Franssen FME , Wouters EFM , Schols AMWJ . A randomized clinical trial investigating the efficacy of targeted nutrition as adjunct to exercise training in COPD. J Cachexia Sarcopenia Muscle 2017;8:748–758. 28608438 10.1002/jcsm.12219 PMC5659064

[59] Beaudart C , Biver E , Reginster JY , Rizzoli R , Rolland Y , Bautmans I , et al. Validation of the SarQoL®, a specific health‐related quality of life questionnaire for Sarcopenia. J Cachexia Sarcopenia Muscle 2017;8:238–244. 27897430 10.1002/jcsm.12149 PMC5377391

[60] Buckinx F , Landi F , Cesari M , Fielding RA , Visser M , Engelke K , et al. Pitfalls in the measurement of muscle mass: a need for a reference standard. J Cachexia Sarcopenia Muscle 2018;9:269–278. 29349935 10.1002/jcsm.12268 PMC5879987

[61] Tieland M , Trouwborst I , Clark BC . Skeletal muscle performance and ageing. J Cachexia Sarcopenia Muscle 2018;9:3–19. 29151281 10.1002/jcsm.12238 PMC5803609

[62] Daly LE , Ní Bhuachalla ÉB , Power DG , Cushen SJ , James K , Ryan AM . Loss of skeletal muscle during systemic chemotherapy is prognostic of poor survival in patients with foregut cancer. J Cachexia Sarcopenia Muscle 2018;9:315–325. 29318756 10.1002/jcsm.12267 PMC5879982

[63] Choi MH , Oh SN , Lee IK , Oh ST , Won DD . Sarcopenia is negatively associated with long‐term outcomes in locally advanced rectal cancer. J Cachexia Sarcopenia Muscle 2018;9:53–59. 28849630 10.1002/jcsm.12234 PMC5803619

[64] Rhee CM , Ahmadi S‐F , Kovesdy CP , Kalantar‐Zadeh K . Low‐protein diet for conservative management of chronic kidney disease: a systematic review and meta‐analysis of controlled trials. J Cachexia Sarcopenia Muscle 2018;9:235–245. 29094800 10.1002/jcsm.12264 PMC5879959

[65] Zhang Z‐K , Li J , Guan D , Liang C , Zhuo Z , Liu J , et al. A newly identified lncRNA MAR1 acts as a miR‐487b sponge to promote skeletal muscle differentiation and regeneration. J Cachexia Sarcopenia Muscle 2018;9:613–626. 29512357 10.1002/jcsm.12281 PMC5989759

[66] Mücke M , Weier M , Carter C , Copeland J , Degenhardt L , Cuhls H , et al. Systematic review and meta‐analysis of cannabinoids in palliative medicine. J Cachexia Sarcopenia Muscle 2018;9:220–234. 29400010 10.1002/jcsm.12273 PMC5879974

[67] Mayr R , Gierth M , Zeman F , Reiffen M , Seeger P , Wezel F , et al. Sarcopenia as a comorbidity‐independent predictor of survival following radical cystectomy for bladder cancer. J Cachexia Sarcopenia Muscle 2018;9:505–513. 29479839 10.1002/jcsm.12279 PMC5989852

[68] Calder PC , Laviano A , Lonnqvist F , Muscaritoli M , Öhlander M , Schols A . Targeted medical nutrition for cachexia in chronic obstructive pulmonary disease: a randomized, controlled trial. J Cachexia Sarcopenia Muscle 2018;9:28–40. 28891198 10.1002/jcsm.12228 PMC5803606

[69] Yang Q‐J , Zhao J‐R , Hao J , Li B , Huo Y , Han Y‐L , et al. Serum and urine metabolomics study reveals a distinct diagnostic model for cancer cachexia. J Cachexia Sarcopenia Muscle 2018;9:71–85. 29152916 10.1002/jcsm.12246 PMC5803608

[70] Zhang A , Li M , Wang B , Klein JD , Price SR , Wang XH . miRNA‐23a/27a attenuates muscle atrophy and renal fibrosis through muscle‐kidney crosstalk. J Cachexia Sarcopenia Muscle 2018;9:755–770. 29582582 10.1002/jcsm.12296 PMC6104113

[71] Connolly M , Paul R , Farre‐Garros R , Natanek SA , Bloch S , Lee J , et al. miR‐424‐5p reduces ribosomal RNA and protein synthesis in muscle wasting. J Cachexia Sarcopenia Muscle 2018;9:400–416. 29215200 10.1002/jcsm.12266 PMC5879973

[72] Paul R , Lee J , Donaldson AV , Connolly M , Sharif M , Natanek SA , et al. miR‐422a suppresses SMAD4 protein expression and promotes resistance to muscle loss. J Cachexia Sarcopenia Muscle 2018;9:119–128. 28984049 10.1002/jcsm.12236 PMC5803610

[73] Ní Bhuachalla ÉB , Daly LE , Power DG , Cushen SJ , MacEneaney P , Ryan AM . Computed tomography diagnosed cachexia and sarcopenia in 725 oncology patients: is nutritional screening capturing hidden malnutrition? J Cachexia Sarcopenia Muscle 2018;9:295–305. 29271097 10.1002/jcsm.12258 PMC5879969

[74] Cala MP , Agulló‐Ortuño MT , Prieto‐García E , González‐Riano C , Parrilla‐Rubio L , Barbas C , et al. Multiplatform plasma fingerprinting in cancer cachexia: a pilot observational and translational study. J Cachexia Sarcopenia Muscle 2018;9:348–357. 29464940 10.1002/jcsm.12270 PMC5879957

[75] Hardee JP , Counts BR , Gao S , VanderVeen BN , Fix DK , Koh H‐J , et al. Inflammatory signalling regulates eccentric contraction‐induced protein synthesis in cachectic skeletal muscle. J Cachexia Sarcopenia Muscle 2018;9:369–383. 29215198 10.1002/jcsm.12271 PMC5879978

[76] Nissinen TA , Hentilä J , Penna F , Lampinen A , Lautaoja JH , Fachada V , et al. Treating cachexia using soluble ACVR2B improves survival, alters mTOR localization, and attenuates liver and spleen responses. J Cachexia Sarcopenia Muscle 2018;9:514–529. 29722201 10.1002/jcsm.12310 PMC5989872

[77] Siracusa J , Koulmann N , Banzet S . Circulating myomiRs: a new class of biomarkers to monitor skeletal muscle in physiology and medicine. J Cachexia Sarcopenia Muscle 2018;9:20–27. 29193905 10.1002/jcsm.12227 PMC5803618

[78] Kays JK , Shahda S , Stanley M , Bell TM , O'Neill BH , Kohli MD , et al. Three cachexia phenotypes and the impact of fat‐only loss on survival in FOLFIRINOX therapy for pancreatic cancer. J Cachexia Sarcopenia Muscle 2018;9:673–684. 29978562 10.1002/jcsm.12307 PMC6104116

[79] Talbert EE , Lewis HL , Farren MR , Ramsey ML , Chakedis JM , Rajasekera P , et al. Circulating monocyte chemoattractant protein‐1 (MCP‐1) is associated with cachexia in treatment‐naïve pancreatic cancer patients. J Cachexia Sarcopenia Muscle 2018;9:358–368. 29316343 10.1002/jcsm.12251 PMC5879958

[80] Ebadi M , Wang CW , Lai JC , Dasarathy S , Kappus MR , Dunn MA , et al. Poor performance of psoas muscle index for identification of patients with higher waitlist mortality risk in cirrhosis. J Cachexia Sarcopenia Muscle 2018;9:1053–1062. 30269421 10.1002/jcsm.12349 PMC6240754

[81] Golan T , Geva R , Richards D , Madhusudan S , Lin BK , Wang HT , et al. LY2495655, an antimyostatin antibody, in pancreatic cancer: a randomized, phase 2 trial. J Cachexia Sarcopenia Muscle 2018;9:871–879. 30051975 10.1002/jcsm.12331 PMC6204586

[82] Pijl R , Strom J , Conijn S , Lindqvist J , Labeit S , Granzier H , et al. Titin‐based mechanosensing modulates muscle hypertrophy. J Cachexia Sarcopenia Muscle 2018;9:947–961. 29978560 10.1002/jcsm.12319 PMC6204599

[83] Peng L‐N , Lee W‐J , Liu L‐K , Lin M‐H , Chen L‐K . Healthy community‐living older men differ from women in associations between myostatin levels and skeletal muscle mass. J Cachexia Sarcopenia Muscle 2018;9:635–642. 29654636 10.1002/jcsm.12302 PMC6104118

[84] Shankaran M , Czerwieniec G , Fessler C , Wong P‐A , Killion S , Turner SM , et al. Dilution of oral D3‐Creatine to measure creatine pool size and estimate skeletal muscle mass: development of a correction algorithm. J Cachexia Sarcopenia Muscle 2018;9:540–546. 29663711 10.1002/jcsm.12278 PMC5989770

[85] Akkasheh G , Kashani‐Poor Z , Tajabadi‐Ebrahimi M , Jafari P , Akbari H , Taghizadeh M , et al. Clinical and metabolic response to probiotic administration in patients with major depressive disorder: a randomized, double‐blind, placebo‐controlled trial. Nutrition 2016;32:315–320. 26706022 10.1016/j.nut.2015.09.003

[86] Diaz‐Gerevini G , Repossi G , Dain A , Tarres M , Das U , Eynard A . Beneficial action of resveratrol: how and why? Nutrition 2016;32:174–178. 26706021 10.1016/j.nut.2015.08.017

[87] Sahebkar A , Serban M , Gluba‐Brzózka A , Mikhailidis D , Cicero A , Rysz J , et al. Lipid‐modifying effects of nutraceuticals: an evidence‐based approach. Nutrition 2016;32:1179–1192. 27324061 10.1016/j.nut.2016.04.007

[88] Liu X , Yan Y , Li F , Zhang D . Fruit and vegetable consumption and the risk of depression: a meta‐analysis. Nutrition 2016;32:296–302. 26691768 10.1016/j.nut.2015.09.009

[89] Hamaguchi Y , Kaido T , Okumura S , Kobayashi A , Hammad A , Tamai Y , et al. Proposal for new diagnostic criteria for low skeletal muscle mass based on computed tomography imaging in Asian adults. Nutrition 2016;32:1200–1205. 27292773 10.1016/j.nut.2016.04.003

[90] Obih C , Wahbeh G , Lee D , Braly K , Giefer M , Shaffer M , et al. Specific carbohydrate diet for pediatric inflammatory bowel disease in clinical practice within an academic IBD center. Nutrition 2016;32:418–425. 26655069 10.1016/j.nut.2015.08.025

[91] Venturelli S , Burkard M , Biendl M , Lauer U , Frank J , Busch C . Prenylated chalcones and flavonoids for the prevention and treatment of cancer. Nutrition 2016;32:1171–1178. 27238957 10.1016/j.nut.2016.03.020

[92] Sadeghian M , Saneei P , Siassi F , Esmaillzadeh A . Vitamin D status in relation to Crohn's disease: meta‐analysis of observational studies. Nutrition 2016;32:505–514. 26837598 10.1016/j.nut.2015.11.008

[93] Thomas M , Kufeldt J , Kisser U , Hornung H , Hoffmann J , Andraschko M , et al. Effects of malnutrition on complication rates, length of hospital stay, and revenue in elective surgical patients in the G‐DRG‐system. Nutrition 2016;32:249–254. 26688128 10.1016/j.nut.2015.08.021

[94] Panahi Y , Hosseini M , Khalili N , Naimi E , Soflaei S , Majeed M , et al. Effects of supplementation with curcumin on serum adipokine concentrations: a randomized controlled trial. Nutrition 2016;32:1116–1122. 27297718 10.1016/j.nut.2016.03.018

[95] Kashtanova D , Popenko A , Tkacheva O , Tyakht A , Alexeev D , Boytsov S . Association between the gut microbiota and diet: fetal life, early childhood, and further life. Nutrition 2016;32:620–627. 26946974 10.1016/j.nut.2015.12.037

[96] Rouhani M , Haghighatdoost F , Surkan P , Azadbakht L . Associations between dietary energy density and obesity: a systematic review and meta‐analysis of observational studies. Nutrition 2016;32:1037–1047. 27238958 10.1016/j.nut.2016.03.017

[97] Sarrafzadegan N , Khosravi‐Boroujeni H , Lotfizadeh M , Pourmogaddas A , Salehi‐Abargouei A . Magnesium status and the metabolic syndrome: a systematic review and meta‐analysis. Nutrition 2016;32:409–417. 26919891 10.1016/j.nut.2015.09.014

[98] Yamagishi S , Matsui T . Pathologic role of dietary advanced glycation end products in cardiometabolic disorders, and therapeutic intervention. Nutrition 2016;32:157–165. 26602289 10.1016/j.nut.2015.08.001

[99] Sahebkar A , Serban C , Ursoniu S , Banach M . Effect of garlic on plasma lipoprotein(a) concentrations: a systematic review and meta‐analysis of randomized controlled clinical trials. Nutrition 2016;32:33–40. 26522661 10.1016/j.nut.2015.06.009

[100] Rincón‐Cervera M , Valenzuela R , Hernandez‐Rodas M , Marambio M , Espinosa A , Mayer S , et al. Supplementation with antioxidant‐rich extra virgin olive oil prevents hepatic oxidative stress and reduction of desaturation capacity in mice fed a high‐fat diet: effects on fatty acid composition in liver and extrahepatic tissues. Nutrition 2016;32:1254–1267. 27346714 10.1016/j.nut.2016.04.006

[101] Bernini L , Simão A , Alfieri D , Lozovoy M , Mari N , de Souza C , et al. Beneficial effects of Bifidobacterium lactis on lipid profile and cytokines in patients with metabolic syndrome: a randomized trial. Effects of probiotics on metabolic syndrome. Nutrition 2016;32:716–719. 27126957 10.1016/j.nut.2015.11.001

[102] Manna P , Kalita J . Beneficial role of vitamin K supplementation on insulin sensitivity, glucose metabolism, and the reduced risk of type 2 diabetes: a review. Nutrition 2016;32:732–739. 27133809 10.1016/j.nut.2016.01.011

[103] Schollenberger A , Karschin J , Meile T , Küper M , Königsrainer A , Bischoff S . Impact of protein supplementation after bariatric surgery: a randomized controlled double‐blind pilot study. Nutrition 2016;32:186–192. 26691769 10.1016/j.nut.2015.08.005

[104] Bounoure L , Gomes F , Stanga Z , Keller U , Meier R , Ballmer P , et al. Detection and treatment of medical inpatients with or at‐risk of malnutrition: suggested procedures based on validated guidelines. Nutrition 2016;32:790–798. 27160498 10.1016/j.nut.2016.01.019

[105] Sandini M , Bernasconi D , Fior D , Molinelli M , Ippolito D , Nespoli L , et al. A high visceral adipose tissue‐to‐skeletal muscle ratio as a determinant of major complications after pancreatoduodenectomy for cancer. Nutrition 2016;32:1231–1237. 27261062 10.1016/j.nut.2016.04.002

[106] Marques‐Rocha J , Milagro F , Mansego M , Zulet M , Bressan J , Martínez J . Expression of inflammation‐related miRNAs in white blood cells from subjects with metabolic syndrome after 8 wk of following a Mediterranean diet‐based weight loss program. Nutrition 2016;32:48–55. 26421388 10.1016/j.nut.2015.06.008

[107] Caccialanza R , Cereda E , Pinto C , Cotogni P , Farina G , Gavazzi C , et al. Awareness and consideration of malnutrition among oncologists: insights from an exploratory survey. Nutrition 2016;32:1028–1032. 27066746 10.1016/j.nut.2016.02.005

[108] Silvester J , Weiten D , Graff L , Walker J , Duerksen D . Is it gluten‐free? Relationship between self‐reported gluten‐free diet adherence and knowledge of gluten content of foods. Nutrition 2016;32:777–783. 27131408 10.1016/j.nut.2016.01.021 PMC5457910

[109] Alvarez J , Ziegler T , Millson E , Stecenko A . Body composition and lung function in cystic fibrosis and their association with adiposity and normal‐weight obesity. Nutrition 2016;32:447–452. 26740256 10.1016/j.nut.2015.10.012 PMC4769897

[110] Skalickova S , Milosavljevic V , Cihalova K , Horky P , Richtera L , Adam V . Selenium nanoparticles as a nutritional supplement. Nutrition 2017;33:83–90. 27356860 10.1016/j.nut.2016.05.001

[111] Bjørklund G , Chirumbolo S . Role of oxidative stress and antioxidants in daily nutrition and human health. Nutrition 2017;33:311–321. 27746034 10.1016/j.nut.2016.07.018

[112] Sharma K , Mahato N , Cho M , Lee Y . Converting citrus wastes into value‐added products: economic and environmently friendly approaches. Nutrition 2017;34:29–46. 28063510 10.1016/j.nut.2016.09.006

[113] Friedli N , Stanga Z , Sobotka L , Culkin A , Kondrup J , Laviano A , et al. Revisiting the refeeding syndrome: results of a systematic review. Nutrition 2017;35:151–160. 28087222 10.1016/j.nut.2016.05.016

[114] DeBoer M , Scharf R , Leite A , Férrer A , Havt A , Pinkerton R , et al. Systemic inflammation, growth factors, and linear growth in the setting of infection and malnutrition. Nutrition 2017;33:248–253. 27712965 10.1016/j.nut.2016.06.013 PMC5193489

[115] Kaido T , Tamai Y , Hamaguchi Y , Okumura S , Kobayashi A , Shirai H , et al. Effects of pretransplant sarcopenia and sequential changes in sarcopenic parameters after living donor liver transplantation. Nutrition 2017;33:195–198. 27649861 10.1016/j.nut.2016.07.002

[116] Farinetti A , Zurlo V , Manenti A , Coppi F , Mattioli A . Mediterranean diet and colorectal cancer: a systematic review. Nutrition 2017;43‐44:83–88. 10.1016/j.nut.2017.06.008 28935150

[117] Muros J , Cofre‐Bolados C , Arriscado D , Zurita F , Knox E . Mediterranean diet adherence is associated with lifestyle, physical fitness, and mental wellness among 10‐y‐olds in Chile. Nutrition 2017;35:87–92. 28241995 10.1016/j.nut.2016.11.002

[118] Sur S , Panda C . Molecular aspects of cancer chemopreventive and therapeutic efficacies of tea and tea polyphenols. Nutrition 2017;43‐44:8–15. 10.1016/j.nut.2017.06.006 28935149

[119] Eglseer D , Halfens R , Lohrmann C . Is the presence of a validated malnutrition screening tool associated with better nutritional care in hospitalized patients? Nutrition 2017;37:104–111. 28359355 10.1016/j.nut.2016.12.016

[120] Charytoniuk T , Drygalski K , Konstantynowicz‐Nowicka K , Berk K , Chabowski A . Alternative treatment methods attenuate the development of NAFLD: a review of resveratrol molecular mechanisms and clinical trials. Nutrition 2017;34:108–117. 28063505 10.1016/j.nut.2016.09.001

[121] Akhtar N , Khan N , Ashruf O , Haqqi T . Inhibition of cartilage degradation and suppression of PGE2 and MMPs expression by pomegranate fruit extract in a model of posttraumatic osteoarthritis. Nutrition 2017;33:1–13. 27908544 10.1016/j.nut.2016.08.004 PMC5137805

[122] Holeček M . Branched‐chain amino acid supplementation in treatment of liver cirrhosis: updated views on how to attenuate their harmful effects on cataplerosis and ammonia formation. Nutrition 2017;41:80–85. 28760433 10.1016/j.nut.2017.04.003

[123] Gundala N , Naidu V , Das U . Arachidonic acid and lipoxinA4 attenuate streptozotocin‐induced cytotoxicity to RIN5 F cells in vitro and type 1 and type 2 diabetes mellitus in vivo. Nutrition 2017;35:61–80. 28241993 10.1016/j.nut.2016.10.004

[124] Tang Y , Wu Y , Huang Z , Dong W , Deng Y , Wang F , et al. Administration of probiotic mixture DM#1 ameliorated 5‐fluorouracil–induced intestinal mucositis and dysbiosis in rats. Nutrition 2017;33:96–104. 27427511 10.1016/j.nut.2016.05.003

[125] Abdulrazaq M , Innes J , Calder P . Effect of ω‐3 polyunsaturated fatty acids on arthritic pain: a systematic review. Nutrition 2017;39‐40:57–66. 10.1016/j.nut.2016.12.003 28606571

[126] Della Corte C , Mosca A , Vania A , Alterio A , Iasevoli S , Nobili V . Good adherence to the Mediterranean diet reduces the risk for NASH and diabetes in pediatric patients with obesity: The results of an Italian Study. Nutrition 2017;39‐40:8–14. 10.1016/j.nut.2017.02.008 28606575

[127] Rajizadeh A , Mozaffari‐Khosravi H , Yassini‐Ardakani M , Dehghani A . Effect of magnesium supplementation on depression status in depressed patients with magnesium deficiency: a randomized, double‐blind, placebo‐controlled trial. Nutrition 2017;35:56–60. 28241991 10.1016/j.nut.2016.10.014

[128] Karuppagounder V , Arumugam S , Giridharan V , Sreedhar R , Bose R , Vanama J , et al. Tiny molecule, big power: multi‐target approach for curcumin in diabetic cardiomyopathy. Nutrition 2017;34:47–54. 28063511 10.1016/j.nut.2016.09.005

[129] Han S , Jiao J , Zhang W , Xu J , Zhang W , Fu C , et al. Lipolysis and thermogenesis in adipose tissues as new potential mechanisms for metabolic benefits of dietary fiber. Nutrition 2017;33:118–124. 27461561 10.1016/j.nut.2016.05.006

[130] Molin Netto B , Earthman C , Farias G , Landi Masquio D , Grotti Clemente A , Peixoto P , et al. Eating patterns and food choice as determinant of weight loss and improvement of metabolic profile after RYGB. Nutrition 2017;33:125–131. 27474230 10.1016/j.nut.2016.05.007

[131] Cruz K , de Oliveira A , Morais J , Severo J , Marreiro PD , D. Role of microRNAs on adipogenesis, chronic low‐grade inflammation, and insulin resistance in obesity. Nutrition 2017;35:28–35. 28241987 10.1016/j.nut.2016.10.003

[132] Clayton Z , Fusco E , Kern M . Egg consumption and heart health: a review. Nutrition 2017;37:79–85. 28359368 10.1016/j.nut.2016.12.014

[133] Bhaswant M , Shafie S , Mathai M , Mouatt P , Brown L . Anthocyanins in chokeberry and purple maize attenuate diet‐induced metabolic syndrome in rats. Nutrition 2017;41:24–31. 28760424 10.1016/j.nut.2016.12.009

[134] Aoe S , Ichinose Y , Kohyama N , Komae K , Takahashi A , Abe D , et al. Effects of high β‐glucan barley on visceral fat obesity in Japanese individuals: a randomized, double‐blind study. Nutrition 2017;42:1–6. 28870472 10.1016/j.nut.2017.05.002

[135] Schumann D , Klose P , Lauche R , Dobos G , Langhorst J , Cramer H . Low fermentable, oligo‐, di‐, mono‐saccharides and polyol diet in the treatment of irritable bowel syndrome: a systematic review and meta‐analysis. Nutrition 2018;45:24–31. 29129233 10.1016/j.nut.2017.07.004

[136] Nowiński A , Ufnal M . Trimethylamine N‐oxide: a harmful, protective or diagnostic marker in lifestyle diseases? Nutrition 2018;46:7–12. 29290360 10.1016/j.nut.2017.08.001

[137] Gioxari A , Kaliora A , Marantidou F , Panagiotakos D . Intake of ω‐3 polyunsaturated fatty acids in patients with rheumatoid arthritis: a systematic review and meta‐analysis. Nutrition 2018;45:114–124.e4. 28965775 10.1016/j.nut.2017.06.023

[138] Parker E , Roy T , D'Adamo C , Wieland L . Probiotics and gastrointestinal conditions: an overview of evidence from the Cochrane Collaboration. Nutrition 2018;45:125–134.e11. 28870406 10.1016/j.nut.2017.06.024 PMC5683921

[139] Tewari N , Awad S , Macdonald I , Lobo D . A comparison of three methods to assess body composition. Nutrition 2018;47:1–5. 29429527 10.1016/j.nut.2017.09.005

[140] Mafra D , Borges N , Cardozo L , Anjos J , Black A , Moraes C , et al. Red meat intake in chronic kidney disease patients: two sides of the coin. Nutrition 2018;46:26–32. 29290351 10.1016/j.nut.2017.08.015

[141] Shivappa N , Bonaccio M , Hebert J , Di Castelnuovo A , Costanzo S , Ruggiero E , et al. Association of proinflammatory diet with low‐grade inflammation: results from the Moli‐sani study. Nutrition 2018;54:182–188. 29982145 10.1016/j.nut.2018.04.004 PMC6138548

[142] Gianfredi V , Salvatori T , Nucci D , Villarini M , Moretti M . Can chocolate consumption reduce cardio‐cerebrovascular risk? A systematic review and meta‐analysis. Nutrition 2018;46:103–114. 29290347 10.1016/j.nut.2017.09.006

[143] Zhang N , Ju Z , Zuo T . Time for food: the impact of diet on gut microbiota and human health. Nutrition 2018;51‐52:80–85. 10.1016/j.nut.2017.12.005 29621737

[144] Sampasa‐Kanyinga H , Hamilton H , Chaput J . Sleep duration and consumption of sugar‐sweetened beverages and energy drinks among adolescents. Nutrition 2018;48:77–81. 29469025 10.1016/j.nut.2017.11.013

[145] Thiennimitr P , Yasom S , Tunapong W , Chunchai T , Wanchai K , Pongchaidecha A , et al. Lactobacillus paracasei HII01, xylooligosaccharides, and synbiotics reduce gut disturbance in obese rats. Nutrition 2018;54:40–47. 29705500 10.1016/j.nut.2018.03.005

[146] Pineda‐Juárez J , Lozada‐Mellado M , Ogata‐Medel M , Hinojosa‐Azaola A , Santillán‐Díaz C , Llorente L , et al. Body composition evaluated by body mass index and bioelectrical impedance vector analysis in women with rheumatoid arthritis. Nutrition 2018;53:49–53. 29655777 10.1016/j.nut.2018.01.004

[147] Rinninella E , Persiani R , D'Ugo D , Pennestrì F , Cicchetti A , Di Brino E , et al. NutriCatt protocol in the Enhanced Recovery After Surgery (ERAS) program for colorectal surgery: the nutritional support improves clinical and cost‐effectiveness outcomes. Nutrition 2018;50:74–81. 29547797 10.1016/j.nut.2018.01.013

[148] Bermudes A , de Carvalho W , Zamberlan P , Muramoto G , Maranhão R , Delgado A . Changes in lipid metabolism in pediatric patients with severe sepsis and septic shock. Nutrition 2018;47:104–109. 29429528 10.1016/j.nut.2017.09.015

[149] Mou D , Wang J , Liu H , Chen Y , Che L , Fang Z , et al. Maternal methyl donor supplementation during gestation counteracts bisphenol A‐induced oxidative stress in sows and offspring. Nutrition 2018;45:76–84. 29129240 10.1016/j.nut.2017.03.012

[150] Bielinska K , Radkowski M , Grochowska M , Perlejewski K , Huc T , Jaworska K , et al. High salt intake increases plasma trimethylamine N‐oxide (TMAO) concentration and produces gut dysbiosis in rats. Nutrition 2018;54:33–39. 29705499 10.1016/j.nut.2018.03.004

[151] Reichenberger J , Richard A , Smyth J , Fischer D , Pollatos O , Blechert J . It's craving time: time of day effects on momentary hunger and food craving in daily life. Nutrition 2018;55‐56:15–20. 10.1016/j.nut.2018.03.048 29960151

[152] Brasil G , Silva‐Cutini M , Moraes F , Pereira T , Vasquez E , Lenz D , et al. The benefits of soluble non‐bacterial fraction of kefir on blood pressure and cardiac hypertrophy in hypertensive rats are mediated by an increase in baroreflex sensitivity and decrease in angiotensin‐converting enzyme activity. Nutrition 2018;51‐52:66–72. 10.1016/j.nut.2017.12.007 29605766

[153] Ylinen E , Merras‐Salmio L , Gunnar R , Jahnukainen T , Pakarinen M . Intestinal failure as a significant risk factor for renal impairment in children. Nutrition 2018;45:90–93. 29129242 10.1016/j.nut.2017.07.011

[154] Kim H , Kim Y , Lee E , Huh J , Chung C . Caffeic acid ameliorates hepatic steatosis and reduces ER stress in high fat diet‐induced obese mice by regulating autophagy. Nutrition 2018;55‐56:63–70. 10.1016/j.nut.2018.03.010 29960159

[155] Nunes S , Alves D , Barreto P , Raimundo M , da Luz Cachulo M , Farinha C , et al. Adherence to a Mediterranean diet and its association with age‐related macular degeneration. The Coimbra Eye Study–Report 4. Nutrition 2018;51‐52:6–12. 10.1016/j.nut.2017.12.010 29547735

[156] Moradi S , Issah A , Mohammadi H , Mirzaei K . Associations between dietary inflammatory index and incidence of breast and prostate cancer: a systematic review and meta‐analysis. Nutrition 2018;55‐56:168–178. 10.1016/j.nut.2018.04.018 30086486

[157] Shtriker M , Hahn M , Taieb E , Nyska A , Moallem U , Tirosh O , et al. Fenugreek galactomannan and citrus pectin improve several parameters associated with glucose metabolism and modulate gut microbiota in mice. Nutrition 2018;46:134–142.e3. 28993009 10.1016/j.nut.2017.07.012

[158] Della Valle S , Colatruglio S , La Vela V , Tagliabue E , Mariani L , Gavazzi C . Nutritional intervention in head and neck cancer patients during chemo‐radiotherapy. Nutrition 2018;51‐52:95–97. 10.1016/j.nut.2017.12.012 29625408

[159] Pounis G , Costanzo S , Bonaccio M , Di Castelnuovo A , Curtis A , Ruggiero E , et al. Reduced mortality risk by a polyphenol‐rich diet: an analysis from the Moli‐sani study. Nutrition 2018;48:87–95. 29469027 10.1016/j.nut.2017.11.012

[160] Shimada H , Makizako H , Lee S , Doi T , Tsutsumimoto K , Harada K , et al. Impact of cognitive frailty on daily activities in older persons. J Nutr Health Aging 2016;20:729–735.2749930610.1007/s12603-016-0685-2

[161] Pilgrim A , Baylis D , Jameson K , Cooper C , Sayer AA , Robinson SM , et al. Measuring appetite with the simplified nutritional appetite questionnaire identifies hospitalised older people at risk of worse health outcomes. J Nutr Health Aging 2016;20:3–7. 26728926 10.1007/s12603-016-0668-3 PMC4778266

[162] Boespflug EL , McNamara RK , Eliassen JC , Schidler MD . Fish oil supplementation increases event‐related posterior cingulate activation in older adults with subjective memory impairment. J Nutr Health Aging 2016;20:161–169.2681251210.1007/s12603-015-0609-6

[163] Warnier RMJ , van Rossum E , van Velthuijsen E , Mulder WJ , Schols JM , Kempen GI . Validity, reliability and feasibility of tools to identify frail older patients in inpatient hospital care: A systematic review. J Nutr Health Aging 2016;20:218–230.2681252010.1007/s12603-015-0567-z

[164] Kaehr EW , Pape LC , Malmstrom TK , Morley JE . FRAIL‐NH predicts outcomes in long term care. J Nutr Health Aging 2016;20:192–198.2681251610.1007/s12603-016-0682-5

[165] Yoshimura Y , Uchida K , Jeong S , Yamaga M . Effects of nutritional supplements on muscle mass and activities of daily living in elderly rehabilitation patients with decreased muscle mass: A randomized controlled trial. J Nutr Health Aging 2016;20:185–191.2681251510.1007/s12603-015-0570-4

[166] Blain H , Masud T , Dargent‐Molina P , Martin FC , Rosendahl E , van der Velde N , et al. A comprehensive fracture prevention strategy in older adults: The European Union Geriatric Medicine Society (EUGMS) statement. J Nutr Health Aging 2016;20:647–652. 27273355 10.1007/s12603-016-0741-y PMC5094892

[167] Madhavan A , Lagorio LA , Crary MA , Dahl WJ , Carnaby GD . Prevalence of and risk factors for dysphagia in the community dwelling elderly: A systematic review. J Nutr Health Aging 2016;20:806–815.2770922910.1007/s12603-016-0712-3

[168] Tay L , Lim WS , Chan M , Ye RJ , Chong MS . The independent role of inflammation in physical frailty among older adults with mild cognitive impairment and mild‐to‐moderate Alzheimer's disease. J Nutr Health Aging 2016;20:288–299.2689257810.1007/s12603-015-0617-6

[169] Scott D , Park MS , Kim TN , Ryu JY , Hong HC , Yoo HJ , et al. Associations of low muscle mass and the metabolic syndrome in Caucasian and Asian middle‐aged and older adults. J Nutr Health Aging 2016;20:248–255.2689257310.1007/s12603-015-0559-z

[170] Wakabayashi H , Matsushima M . Dysphagia assessed by the 10‐item eating assessment tool is associated with nutritional status and activities of daily living in elderly individuals requiring long‐term care. J Nutr Health Aging 2016;20:22–27.2672892910.1007/s12603-016-0671-8

[171] Armamento‐Villareal R , Aguirre LE , Qualls C , Villareal DT . Effect of lifestyle intervention on the hormonal profile of frail, obese older men. J Nutr Health Aging 2016;20:334–340. 26892583 10.1007/s12603-016-0698-x PMC4811358

[172] De Vriendt P , Peersman W , Florus A , Verbeke M , Van de Velde D . Improving health related quality of life and independence in community dwelling frail older adults through a client‐centred and activity‐oriented program. A pragmatic randomized controlled trial. J Nutr Health Aging 2016;20:35–40.2672893110.1007/s12603-016-0673-6

[173] de Souza Vasconcelos KS , Domingues Dias JM , de Carvalho Bastone A , Vieira RA , de Souza Andrade AC , Perracini MR , et al. Handgrip strength cutoff points to identify mobility limitation in community‐dwelling older people and associated factors. J Nutr Health Aging 2016;20:306–315.2689258010.1007/s12603-015-0584-y

[174] Molino S , Dossena M , Buonocore D , Verri M . Sarcopenic obesity: An appraisal of the current status of knowledge and management in elderly people. J Nutr Health Aging 2016;20:780–788.2749931210.1007/s12603-015-0631-8

[175] Morilla‐Herrera JC , Martín‐Santos FJ , Caro‐Bautista J , Saucedo‐Figueredo C , Garcia‐Mayor S , Morales‐Asencio JM . Effectiveness of food‐based fortification in older people a systematic review and meta‐analysis. J Nutr Health Aging 2016;20:178–184.2681251410.1007/s12603-015-0591-z

[176] Martínez‐Velilla N , Cadore EL , Casas‐Herrero Á , Idoate‐Saralegui F , Izquierdo M . Physical activity and early rehabilitation in hospitalized elderly medical patients: Systematic review of randomized clinical trials. J Nutr Health Aging 2016;20:738–751.2749930810.1007/s12603-016-0683-4

[177] Fougère B , Mazzuco S , Spagnolo P , Guyonnet S , Vellas B , Cesari M , et al. Association between the Mediterranean‐style dietary pattern score and physical performance: Results from TRELONG study. J Nutr Health Aging 2016;20:415–419.2699924210.1007/s12603-015-0588-7

[178] Abraha I , Rimland JM , Trotta F , Pierini V , Cruz‐Jentoft A , Soiza R , et al. Non‐pharmacological interventions to prevent or treat delirium in older patients: Clinical practice recommendations the SENATOR‐ONTOP series. J Nutr Health Aging 2016;20:927–936.2779122310.1007/s12603-016-0719-9

[179] Hajek A , Brettschneider C , Posselt T , Lange C , Mamone S , Wiese B , et al. Predictors of frailty in old age–results of a longitudinal study. J Nutr Health Aging 2016;20:952–957.2779122610.1007/s12603-015-0634-5

[180] Chode S , Malmstrom TK , Miller DK , Morley JE . Frailty, diabetes, and mortality in middle‐aged African Americans. J Nutr Health Aging 2016;20:854–859.2770923510.1007/s12603-016-0801-3

[181] Hentzien M , Dramé M , Allavena C , Jacomet C , Valantin MA , Cabié A , et al. Impact of age‐related comorbidities on five‐year overall mortality among elderly HIV‐infected patients in the late HAART era — Role of chronic renal disease. J Nutr Health Aging 2016;20:408–414.2699924110.1007/s12603-015-0608-7

[182] Lehtisalo J , Lindström J , Ngandu T , Kivipelto M , Ahtiluoto S , Ilanne‐Parikka P , et al. Association of long‐term dietary fat intake, exercise, and weight with later cognitive function in the Finnish Diabetes Prevention Study. J Nutr Health Aging 2016;20:146–154.2681251010.1007/s12603-015-0565-1

[183] van Wissen J , van Stijn MFM , Doodeman HJ , Houdijk AP . Mini nutritional assessment and mortality after hip fracture surgery in the elderly. J Nutr Health Aging 2016;20:964–968.2779122810.1007/s12603-015-0630-9

[184] Beasley JM , Deierlein AL , Morland KB , Granieri EC , Spark A . Is meeting the recommended dietary allowance (RDA) for protein related to body composition among older adults?: Results from the Cardiovascular Health of Seniors and Built Environment Study. J Nutr Health Aging 2016;20:790–796. 27709227 10.1007/s12603-015-0707-5 PMC5348248

[185] Wirth MD , Shivappa N , Davis L , Hurley TG , Ortaglia A , Drayton R , et al. Construct validation of the Dietary Inflammatory Index among African Americans. J Nutr Health Aging 2017;21:487–491. 28448077 10.1007/s12603-016-0775-1 PMC5547883

[186] Roppolo M , Mulasso A , Rabaglietti E . Cognitive frailty in Italian community‐dwelling older adults: Prevalence rate and its association with disability. J Nutr Health Aging 2017;21:631–636.2853732610.1007/s12603-016-0828-5

[187] Balogun S , Winzenberg T , Wills K , Scott D , Jones G , Aitken D , et al. Prospective associations of low muscle mass and function with 10‐year falls risk, incident fracture and mortality in community‐dwelling older adults. J Nutr Health Aging 2017;21:843–848.2871781610.1007/s12603-016-0843-6

[188] Bousquet J , Bewick M , Cano A , Eklund P , Fico G , Goswami N , et al. Building bridges for innovation in ageing: Synergies between action groups of the EIP on AHA. J Nutr Health Aging 2017;21:92–104.2799985510.1007/s12603-016-0803-1

[189] Zhang YY , Liu W , Zhao TY , Tian HM . Efficacy of omega‐3 polyunsaturated fatty acids supplementation in managing overweight and obesity: A meta‐analysis of randomized clinical trials. J Nutr Health Aging 2017;21:187–192.2811277410.1007/s12603-016-0755-5

[190] Misciagna G , del Pilar Díaz M , Caramia DV , Bonfiglio C , Franco I , Noviello MR , et al. Effect of a low glycemic index Mediterranean diet on non‐alcoholic fatty liver disease. A randomized controlled clinici trial. J Nutr Health Aging 2017;21:404–412.2834656710.1007/s12603-016-0809-8

[191] O'Shea E , Trawley S , Manning E , Barrett A , Browne V , Timmons S . Malnutrition in hospitalised older adults: A multicentre observational study of prevalence, associations and outcomes. J Nutr Health Aging 2017;21:830–836.2871781410.1007/s12603-016-0831-x

[192] Hooper C , de Souto Barreto P , Coley N , Cantet C , Cesari M , Andrieu S , et al. Cognitive changes with omega‐3 polyunsaturated fatty acids in non‐demented older adults with low omega‐3 index. J Nutr Health Aging 2017;21:988–993.2908343910.1007/s12603-017-0957-5

[193] Tieland M , Franssen R , Dullemeijer C , van Dronkelaar C , Kim HK , Ispoglou T , et al. The impact of dietary protein or amino acid supplementation on muscle mass and strength in elderly people: Individual participant data and meta‐analysis of RCT's. J Nutr Health Aging 2017;21:994–1001.2908344010.1007/s12603-017-0896-1

[194] Limongi F , Noale M , Gesmundo A , Crepaldi G , Maggi S , ILSA Working Group . Adherence to the Mediterranean Diet and all‐cause mortality risk in an elderly Italian population: Data from the ILSA study. J Nutr Health Aging 2017;21:505–513.2844808010.1007/s12603-016-0808-9

[195] Masanés F , Rojano‐i‐Luque X , Salvà A , Serra‐Rexach JA , Artaza I , Formiga F , et al. Cut‐off points for muscle mass — not grip strength or gait speed — determine variations in sarcopenia prevalence. J Nutr Health Aging 2017;21:825–829.2871781310.1007/s12603-016-0844-5

[196] Mitchell EL , Davis AT , Brass K , Dendinger M , Barner R , Gharaibeh R , et al. Reduced intestinal motility, mucosal barrier function, and inflammation in aged monkeys. J Nutr Health Aging 2017;21:354–361. 28346561 10.1007/s12603-016-0725-y PMC6057140

[197] Landi F , Calvani R , Tosato M , Martone AM , Picca A , Ortolani E , et al. Animal‐derived protein consumption is associated with muscle mass and strength in community‐dwellers: Results from the Milan Expo survey. J Nutr Health Aging 2017;21:1050–1056.2908344710.1007/s12603-017-0974-4

[198] Amamou T , Normandin E , Pouliot J , Dionne IJ , Brochu M , Riesco E . Effect of a high‐protein energy‐restricted diet combined with resistance training on metabolic profile in older individuals with metabolic impairments. J Nutr Health Aging 2017;21:67–74.2799985210.1007/s12603-016-0760-8

[199] Sargent L , Brown R . Assessing the current state of cognitive frailty: Measurement properties. J Nutr Health Aging 2017;21:152–160.2811276910.1007/s12603-016-0735-9

[200] Iolascon G , Gimigliano R , Bianco M , De Sire A , Moretti A , Giusti A , et al. Are dietary supplements and nutraceuticals effective for musculoskeletal health and cognitive function? A scoping review. J Nutr Health Aging 2017;21:527–538.2844808310.1007/s12603-016-0823-x

[201] García‐Nogueras I , Aranda‐Reneo I , Peña‐Longobardo LM , Oliva‐Moreno J , Abizanda P . Use of health resources and healthcare costs associated with frailty: The FRADEA study. J Nutr Health Aging 2017;21:207–214.2811277810.1007/s12603-016-0727-9

[202] Beelen J , de Roos NM , de Groot LCPGM . Protein enrichment of familiar foods as an innovative strategy to increase protein intake in institutionalized elderly. J Nutr Health Aging 2017;21:173–179.2811277210.1007/s12603-016-0733-y

[203] Fielding RA , Travison TG , Kirn DR , Koochek A , Reid KF , von Berens Å , et al. Effect of structured physical activity and nutritional supplementation on physical function in mobility‐limited older adults: Results from the VIVE2 randomized trial. J Nutr Health Aging 2017;21:936–942. 29083433 10.1007/s12603-017-0936-x PMC6751564

[204] Dyer J , Davison G , Marcora SM , Mauger AR . Effect of a Mediterranean type diet on inflammatory and cartilage degradation biomarkers in patients with osteoarthritis. J Nutr Health Aging 2017;21:562–566. 28448087 10.1007/s12603-016-0806-y PMC5405095

[205] Tucker LA . Consumption of nuts and seeds and telomere length in 5,582 men and women of the National Health and Nutrition Examination Survey (NHANES). J Nutr Health Aging 2017;21:233–240.2824456010.1007/s12603-017-0876-5

[206] Bleijenberg N , Zuithoff NPA , Smith AK , De Wit NJ , Schuurmans MJ . Disability in the individual ADL, IADL, and mobility among older adults: A prospective cohort study. J Nutr Health Aging 2017;21:897–903.2897224210.1007/s12603-017-0891-6

[207] Chassagne P , Ducrotte P , Garnier P , Mathiex‐Fortunet H . Tolerance and long‐term efficacy of polyethylene glycol 4000 (Forlax®) compared to lactulose in elderly patients with chronic constipation. J Nutr Health Aging 2017;21:429–439.2834657010.1007/s12603-016-0762-6

[208] Harada H , Kai H , Niiyama H , Nishiyama Y , Katoh A , Yoshida N , et al. Effectiveness of cardiac rehabilitation for prevention and treatment of sarcopenia in patients with cardiovascular disease ‐ a retrospective cross‐sectional analysis. J Nutr Health Aging 2017;21:449–456.2834657210.1007/s12603-016-0743-9

[209] Ritt M , Schülein S , Lubrich H , Bollheimer LC , Sieber CC , Gassmann KG . High‐technology based gait assessment in frail people: Associations between spatio‐temporal and three‐dimensional gait characteristics with frailty status across four different frailty measures. J Nutr Health Aging 2017;21:346–353.2824457710.1007/s12603-016-0764-4

[210] Dent E , Morley JE , Cruz‐Jentoft AJ , Arai H , Kritchevsky SB , Guralnik J , et al. International Clinical Practice Guidelines for Sarcopenia (ICFSR): Screening, Diagnosis and Management. J Nutr Health Aging 2018;22:1148–1161.3049882010.1007/s12603-018-1139-9

[211] Berendsen AM , Kang JH , Feskens EJM , de Groot CP , Grodstein F , van de Rest O . Association of long‐term adherence to the mind diet with cognitive function and cognitive decline in American women. J Nutr Health Aging 2018;22:222–229.2938084910.1007/s12603-017-0909-0

[212] Marshall S . Why is the Skeleton Still in the Hospital Closet? A Look at the Complex Aetiology of Protein‐Energy Malnutrition and its Implications for the Nutrition Care Team. J Nutr Health Aging 2018;22:26–29.2930041810.1007/s12603-017-0900-9

[213] McCullough J , Keller H . The My Meal Intake Tool (M‐MIT): Validity of a Patient Self‐Assessment for Food and Fluid Intake at a Single Meal. J Nutr Health Aging 2018;22:30–37.2930041910.1007/s12603-016-0859-y

[214] Beaudart C , Rabenda V , Simmons M , Geerinck A , De Carvalho IA , Reginster JY , et al. Effects of Protein, Essential Amino Acids, B‐Hydroxy B‐Methylbutyrate, Creatine, Dehydroepiandrosterone and Fatty Acid Supplementation on Muscle Mass, Muscle Strength and Physical Performance in Older People Aged 60 Years and Over. A Systematic Review of the Literature. J Nutr Health Aging 2018;22:117–130.2930043110.1007/s12603-017-0934-z

[215] Rietman ML , van der A DL , van Oostrom SH , Picavet HS , Dollé ME , Van Steeg H , et al. The Association Between BMI and Different Frailty Domains: A U‐Shaped Curve? J Nutr Health Aging 2018;22:8–15.2930041610.1007/s12603-016-0854-3

[216] Zhao WT , Yang M , Wu HM , Yang L , Zhang XM , Huang Y . Systematic Review and Meta‐Analysis of the Association Between Sarcopenia and Dysphagia. J Nutr Health Aging 2018;22:1003–1009.3027210610.1007/s12603-018-1055-z

[217] Kim J , Lee Y , Won CW , Lee KE , Chon D . Nutritional Status and Frailty in Community‐Dwelling Older Korean Adults: The Korean Frailty and Aging Cohort Study. J Nutr Health Aging 2018;22:774–778.3008021810.1007/s12603-018-1005-9

[218] Wang T , Shen J . Usefulness of Simplified Nutritional Appetite Questionnaire (SNAQ) in Appetite Assessment in Elder Patients with Liver Cirrhosis. J Nutr Health Aging 2018;22:911–915.3027209210.1007/s12603-018-1086-5

[219] Sanz‐Paris A , Camprubi‐Robles M , Lopez‐Pedrosa JM , Pereira SL , Rueda R , Ballesteros‐Pomar MD , et al. Role of Oral Nutritional Supplements Enriched with B‐hydroxy‐B‐Methylbutyrate in Maintaining Muscle Function and Improving Clinical Outcomes in Various Clinical Settings. J Nutr Health Aging 2018;22:664–675. 29806855 10.1007/s12603-018-0995-7 PMC5984960

[220] Yu Y , Zhao Y , Teng F , Li J , Guan Y , Xu J , et al. Berberine Improves Cognitive Deficiency and Muscular Dysfunction via Activation of the AMPK/SIRT1/PGC‐1a Pathway in Skeletal Muscle from Naturally Aging Rats. J Nutr Health Aging 2018;22:710–717.2980686010.1007/s12603-018-1015-7

[221] Pagliai G , Sofi F , Vannetti F , Caiani S , Pasquini G , Lova RM , et al. Mediterranean Diet, Food Consumption and Risk of Late‐Life Depression: The Mugello Study. J Nutr Health Aging 2018;22:569–574.2971775510.1007/s12603-018-1019-3

[222] Muñoz‐González C , Vandenberghe‐Descamps M , Feron G , Canon F , Labouré H , Sulmont‐Rossé C . Association between Salivary Hypofunction and Food Consumption in the Elderlies. A Systematic Literature Review. J Nutr Health Aging 2018;22:407–419.2948435510.1007/s12603-017-0960-x

[223] Hidayat K , Chen GC , Wang Y , Zhang Z , Dai X , Szeto IM , et al. Effects of milk proteins supplementation in older adults undergoing resistance training: A meta‐analysis of randomized control trials. J Nutr Health Aging 2018;22:237–245.2938085110.1007/s12603-017-0899-y

[224] Nowson CA , Service C , Appleton J , Grieger JA . The impact of dietary factors on indices of chronic disease in older people: A systematic review. J Nutr Health Aging 2018;22:282–296.2938085710.1007/s12603-017-0920-5

[225] Eglseer D , Halfens RJG , Schols JMGA , Lohrmann C . Dysphagia in Hospitalized Older Patients: Associated Factors and Nutritional Interventions. J Nutr Health Aging 2018;22:103–110.2930042910.1007/s12603-017-0928-x

[226] Derstine BA , Holcombe SA , Goulson RL , Ross BE , Wang NC , Sullivan JA , et al. Quantifying Sarcopenia Reference Values Using Lumbar and Thoracic Muscle Areas in a Healthy Population. J Nutr Health Aging 2018;22:180–185.10.1007/s12603-017-0983-329300439

[227] El Hajj C , Chardigny JM , Boirie Y , Yammine K , Helou M , Walrand S . Effect of Vitamin D Treatment on Glucose Homeostasis and Metabolism in Lebanese Older Adults: A Randomized Controlled Trial. J Nutr Health Aging 2018;22:1128–1132.3037931410.1007/s12603-018-1083-8

[228] Rodríguez Mañas L , García‐Sánchez I , Hendry A , Bernabei R , Roller‐Wirnsberger R , Gabrovec B , et al. Key Messages for a Frailty Prevention and Management Policy in Europe from the Advantage Joint Action Consortium. J Nutr Health Aging 2018;22:892–897.3027208910.1007/s12603-018-1064-y

[229] Acar Tek N , Karaçil‐Ermumcu MŞ . Determinants of Health Related Quality of Life in Home Dwelling Elderly Population: Appetite and Nutritional Status. J Nutr Health Aging 2018;22:996–1002.3027210510.1007/s12603-018-1066-9

[230] Palmer K , Vetrano DL , Marengoni A , Tummolo AM , Villani ER , Acampora N , et al. The Relationship Between Anaemia and Frailty: A Systematic Review and Meta‐Analysis of Observational Studies. J Nutr Health Aging 2018;22:965–974.3027210110.1007/s12603-018-1049-x

[231] Rodriguez‐Rejon AI , Artacho R , Puerta A , Puerta A , Zuñiga A , Ruiz‐Lopez MD . Diagnosis of Sarcopenia in Long‐Term Care Homes for the Elderly: The Sensitivity and Specificity of Two Simplified Algorithms with Respect to the EWGSOP Consensus. J Nutr Health Aging 2018;22:796–801.3008022210.1007/s12603-018-1004-x

[232] Payne M , Morley JE . Dysphagia, Dementia and Frailty. J Nutr Health Aging 2018;22:562–565.2971775310.1007/s12603-018-1033-5

[233] Wang Y , Hao Q , Su L , Liu Y , Liu S , Dong B . Adherence to the Mediterranean Diet and the Risk of Frailty in Old People: A Systematic Review and Meta‐Analysis. J Nutr Health Aging 2018;22:613–618.2971776210.1007/s12603-018-1020-x

[234] Lim SER , Ibrahim K , Sayer AA , Roberts HC . Assessment of Physical Activity of Hospitalised Older Adults: A Systematic Review. J Nutr Health Aging 2018;22:377–386.2948435110.1007/s12603-017-0931-2

[235] von Haehling S , Anker SD . Cachexia as a major underestimated and unmet medical need: facts and numbers. J Cachexia Sarcopenia Muscle 2010;1:1–5. 21475699 10.1007/s13539-010-0002-6 PMC3060651

[236] Dalton JT , Barnette KG , Bohl CE , Hancock ML , Rodriguez D , Dodson ST , et al. The selective androgen receptor modulator GTx‐024 (enobosarm) improves lean body mass and physical function in healthy elderly men and postmenopausal women: results of a double‐blind, placebo‐controlled phase II trial. J Cachexia Sarcopenia Muscle 2011;2:153–161. 22031847 10.1007/s13539-011-0034-6 PMC3177038

[237] Morley JE , Anker SD , von Haehling S . Prevalence, incidence, and clinical impact of sarcopenia: facts, numbers, and epidemiology—update 2014. J Cachexia Sarcopenia Muscle 2014;5:253–259. 25425503 10.1007/s13539-014-0161-y PMC4248415

[238] Fanzani A , Conraads VM , Penna F , Martinet W . Molecular and cellular mechanisms of skeletal muscle atrophy: an update. J Cachexia Sarcopenia Muscle 2012;3:163–179. 22673968 10.1007/s13539-012-0074-6 PMC3424188

[239] Cesari M , Fielding RA , Pahor M , Goodpaster B , Hellerstein M , Kan V , et al. Biomarkers of sarcopenia in clinical trials—recommendations from the International Working Group on Sarcopenia. J Cachexia Sarcopenia Muscle 2012;3:181–190. 22865205 10.1007/s13539-012-0078-2 PMC3424187

[240] Bowen TS , Schuler G , Adams V . Skeletal muscle wasting in cachexia and sarcopenia: molecular pathophysiology and impact of exercise training. J Cachexia Sarcopenia Muscle 2015;6:197–207. 26401465 10.1002/jcsm.12043 PMC4575550

[241] Wakabayashi H , Sakuma K . Rehabilitation nutrition for sarcopenia with disability: a combination of both rehabilitation and nutrition care management. J Cachexia Sarcopenia Muscle 2014;5:269–277. 25223471 10.1007/s13539-014-0162-x PMC4248414

[242] Morley JE , von Haehling S , Anker SD , Vellas B . From sarcopenia to frailty: a road less traveled. J Cachexia Sarcopenia Muscle 2014;5:5–8. 24526568 10.1007/s13539-014-0132-3 PMC3953315

[243] Elkina Y , von Haehling S , Anker SD , Springer J . The role of myostatin in muscle wasting: an overview. J Cachexia Sarcopenia Muscle 2011;2:143–151. 21966641 10.1007/s13539-011-0035-5 PMC3177043

[244] von Haehling S , Morley JE , Anker SD . An overview of sarcopenia: facts and numbers on prevalence and clinical impact. J Cachexia Sarcopenia Muscle 2010;1:129–133. 21475695 10.1007/s13539-010-0014-2 PMC3060646

[245] Fang YZ , Yang S , Wu G . Free radicals, antioxidants, and nutrition. Nutrition 2002;18:872–879. 12361782 10.1016/s0899-9007(02)00916-4

[246] Vellas B , Guigoz Y , Garry P , Nourhashemi F , Bennahum D , Lauque S , et al. The mini nutritional assessment (MNA) and its use in grading the nutritional state of elderly patients. Nutrition 1999;15:116–122. 9990575 10.1016/s0899-9007(98)00171-3

[247] Dubois D , Dubois EF . Nutrition Metabolism Classic—A formula to estimate the approximate surface‐area if height and weight be known (Reprinted from archives internal medicine, vol 17, PG 863, 1916). Nutrition 1989;5:303–311. 2520314

[248] Torres S , Nowson C . Relationship between stress, eating behavior, and obesity. Nutrition 2007;23:887–894. 17869482 10.1016/j.nut.2007.08.008

[249] Kuhajda F . Fatty‐acid synthase and human cancer: new perspectives on its role in tumor biology. Nutrition 2000;16:202–208. 10705076 10.1016/s0899-9007(99)00266-x

[250] Das U . Is obesity an inflammatory condition? Nutrition 2001;17:953–966. 11744348 10.1016/s0899-9007(01)00672-4

[251] Waterland R , Jirtle R . Early nutrition, epigenetic changes at transposons and imprinted genes, and enhanced susceptibility to adult chronic diseases. Nutrition 2004;20:63–68. 14698016 10.1016/j.nut.2003.09.011

[252] Slavin J . Dietary fiber and body weight. Nutrition 2005;21:411–418. 15797686 10.1016/j.nut.2004.08.018

[253] Barker D . Maternal nutrition, fetal nutrition, and disease in later life. Nutrition 1997;13:807–813. 9290095 10.1016/s0899-9007(97)00193-7

[254] Scalzo J , Politi A , Pellegrini N , Mezzetti B , Battino M . Plant genotype affects total antioxidant capacity and phenolic contents in fruit. Nutrition 2005;21:207–213. 15723750 10.1016/j.nut.2004.03.025

[255] Abellan Van Kan G , Rolland Y , Andrieu S , Bauer J , Beauchet O , Bonnefoy M , et al. Gait speed at usual pace as a predictor of adverse outcomes in community‐dwelling older people an International Academy on Nutrition and Aging (IANA) Task Force. J Nutr Health Aging 2009;13:881–889.1992434810.1007/s12603-009-0246-z

[256] Guigoz Y . The Mini Nutritional Assessment (MNA®) Review of the literature‐What does it tell us? J Nutr Health Aging 2006;10:466. 17183419

[257] Kaiser MJ , Bauer JM , Ramsch C , Uter W , Guigoz Y , Cederholm T , et al. Validation of the Mini Nutritional Assessment short‐form (MNA®‐SF): A practical tool for identification of nutritional status. J Nutr Health Aging 2009;13:782–788.1981286810.1007/s12603-009-0214-7

[258] Van Kan GA , Rolland Y , Bergman H , Morley JE , Kritchevsky SB , Vellas B . The I.A.N.A. task force on frailty assessment of older people in clinical practice. J Nutr Health Aging 2008;12:29–37.1816584210.1007/BF02982161

[259] Rolland Y , Czerwinski S , van Kan GA , Morley JE , Cesari M , Onder G , et al. Sarcopenia: Its assessment, etiology, pathogenesis, consequences and future perspectives. J Nutr Health Aging 2008;12:433–450. 18615225 10.1007/BF02982704 PMC3988678

[260] Vellas B , Villars H , Abellani G , Soto ME , Rolland Y , Guigoz Y , et al. Overview of the MNA®‐Its history and challenges. J Nutr Health Aging 2006;10:456. 17183418

[261] Morley JE , Malmstrom TK , Miller DK . A simple frailty questionnaire (FRAIL) predicts outcomes in middle aged African Americans. J Nutr Health Aging 2012;16:601–608. 22836700 10.1007/s12603-012-0084-2 PMC4515112

[262] Bourre JM . Effects of nutrients (in food) on the structure and function of the nervous system: update on dietary requirements for brain. Part 1: micronutrients. J Nutr Health Aging 2006;10:377. 17066209

[263] Jugdaohsingh R . Silicon and bone health. J Nutr Health Aging 2007;11:99. 17435952 PMC2658806

[264] Kelaiditi E , Cesari M , Canevelli M , van Kan GA , Ousset PJ , Gillette‐Guyonnet S , et al. Cognitive frailty: rational and definition from an (I.A.N.A./I.A.G.G.) international consensus group. J Nutr Health Aging 2013;17:726–734.2415464210.1007/s12603-013-0367-2

[265] www.scopus.com.

